# A review of microplastic contamination in the cryosphere

**DOI:** 10.1016/j.isci.2025.114414

**Published:** 2025-12-13

**Authors:** Irteza Qayoom, Faisal Zahoor Jan, Irfan Rashid, Gulzar A. Bhat, Anoop Ambili, Chandan Sarangi

**Affiliations:** 1 Centre for Interdisciplinary Research and Innovations, University of Kashmir, Srinagar, Jammu and Kashmir 190006, India; 2 Department of Geoinformatics, University of Kashmir, Srinagar, Jammu and Kashmir 190006, India; 3 Department of Chemistry, University of Kashmir, Srinagar, Jammu and Kashmir 190006, India; 4 Department of Earth and Environmental Sciences, Indian Institute of Science Education & Research Mohali, Mohali, Punjab 140306, India; 5 Department of Civil Engineering, Indian Institute of Technology Madras, Chennai, Tamil Nadu 600036, India

**Keywords:** Glacial landscapes, Environmental science, Pollution

## Abstract

Microplastics, recognized as an emerging and pervasive environmental contaminant, have been detected in remote cryospheric regions such as Antarctica, Arctic, Andes, Alps, and High Asia. This review synthesizes current knowledge on microplastic occurrence, sources, and transport pathways across cryospheric environments. Microplastics are transported primarily by atmospheric and hydrological processes and accumulate in snow and ice, where they act as temporary sinks. In Polar regions, sea ice plays a vital role in the storage and periodic release of microplastics, whereas high-altitude mountain systems receive significant inputs from mid-latitude emissions. The review also highlights the potential impacts of microplastics on cryospheric systems, including their role in the reduction of albedo, the acceleration of snow and ice melt, and their effects on biodiversity through ecological and trophic disturbances. Key methodological challenges in sampling, extraction, and identification point to the urgent need for standardized protocols. Overall, this review underscores the scientific significance of studying microplastic pollution in the cryosphere and offers directions for future research to improve monitoring, modeling, and impact assessment.

## Graphical abstract

**Figure:**
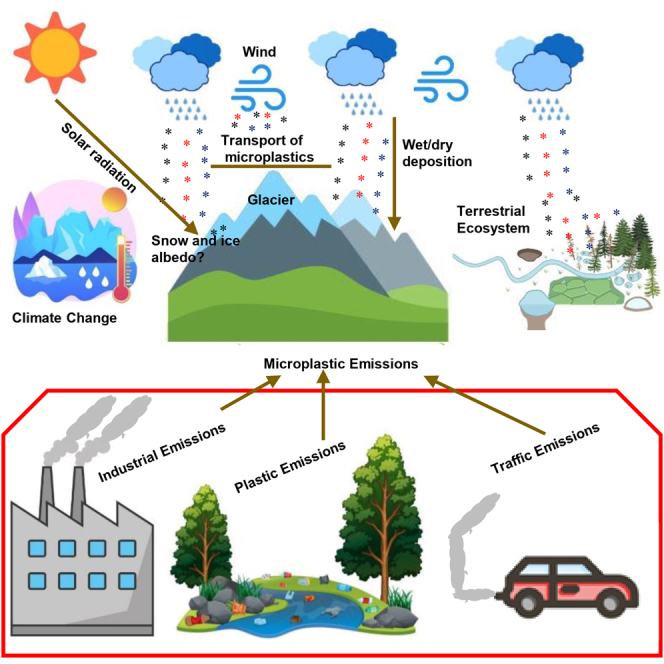

## Introduction

The Anthropocene epoch is characterized by the profound and persistent influence of human activities on the Earth system.^1^^,^^2^^,^^3^^,^^4^ One of the most notable and concerning manifestations of this impact is the substantial rise in plastic production, usage, and inadequate waste management practices.^5^^,^^6^ Plastic pollution is a global concern due to its widespread abundance and persistence in the environment. Global plastic production has experienced exponential growth, increasing from 1.5 million metric tonnes (Mt) in the 1950s to 413.8 million Mt in 2023.^7^ Projections indicate that by 2050, 1.1 billion tonnes of plastic waste could persist in landfills or leak into habitats, far exceeding the 4.9 billion tons recorded in 2015.^8^^,^^9^ Globally, only 9% of plastic waste is recycled, 12% incinerated, while nearly 79% accumulates in various ecosystems.^10^ This accumulated plastic waste is ubiquitous, infiltrating terrestrial and aquatic ecosystems,^11^^,^^12^^,^^13^^,^^14^ and has resulted in significant socio-economic, environmental, and health consequences, prompting significant attention among the scientific community, policymakers, and the public.

Of particular concern is the contribution of plastic waste to an increase in micro-sized particles called microplastic particles (MPs) in the environment, which are broken plastic particles with a size range of 1 μm–5 mm.^15^ These MPs comprise a broad range of polymers with distinct structural and chemical properties,^16^ resulting in different shapes, colors, and sizes. Based on the origin, MPs are categorized as primary and secondary types. Primary MPs are virgin or newly manufactured plastics that have not been recycled or processed before. They are produced directly from petrochemical sources and are used in various industries for packaging, construction, and consumer goods. These include polyethylene (PE), polypropylene (PP), polyvinyl chloride (PVC), polystyrene (PS), and polyethylene terephthalate (PET). Secondary MPs are tiny plastic particles that result from the breakdown of larger plastic items due to various environmental factors such as sunlight, heat, and mechanical abrasion. These MPs are not intentionally manufactured; rather, they result from the degradation and breakdown of larger plastics over time. These include fragments from plastic bags and bottles, synthetic fibers from clothing, tire wear particles, paint chips, fishing nets, and associated gear.^17^^,^^18^ Since the 21^st^ century, MPs have been considered an emerging anthropogenic pollutant and pose a serious global environmental concern^19^^,^^20^^,^^21^^,^^22^ with their presence reported in the oceans,^23^ soil,^24^ rivers,^25^ lakes,^26^ atmosphere,^17^^,^^27^ wetlands,^28^ and groundwater.^29^ MPs also act as carriers for the adsorption and mobility of different types of nutrients, persistent organic pollutants (POPs), heavy metals, and polycyclic aromatic hydrocarbons (PAHs),^30^^,^^31^^,^^32^ thus facilitating their transport across different ecosystems and enhancing pollutant bioavailability.^33^^,^^34^^,^^35^

The cryosphere, which encompasses the frozen landscapes including continental ice sheets, polar ice caps, mountain glaciers, snow, and permafrost, plays a vital role in regulating the global climate system. Recent studies have detected pervasive MPs across the pristine cryospheric environments.^36^^,^^37^^,^^38^^,^^39^ Climate-driven cryosphere degradation, including accelerated ice melt and permafrost thaw, is now exacerbating MP interactions with downstream ecosystems. For instance, melting glaciers and snowpacks may act as secondary MP sources, releasing embedded particles into rivers and oceans,^38^^,^^40^ while thawing permafrost mobilizes legacy plastics and adsorbed pollutants (e.g., heavy metals, POPs) into ecosystems.^41^^,^^42^ These processes pose the risk of amplifying ecological threats, such as bioaccumulation within food chains and food webs^43^^,^^44^, and may alter cryosphere-albedo feedback mechanisms.^36^^,^^45^

Despite growing recognition of MP pollution as a global crisis,^46^ research on its prevalence, sources, and ecological consequences in the cryosphere remains in its infancy. While the sources of MPs are well established in terrestrial and marine ecosystems, their transport and deposition in polar and high-altitude non-polar regions have received far less attention. Studies have shown that MPs can be transported over long distances through atmospheric deposition and ocean currents, eventually settling on glaciers and ice fields, where they accumulate over time.^39^^,^^47^^,^^48^ However, the precise deposition mechanism, the scale of contamination, and the subsequent environmental, biological, and human-health impacts are still poorly understood. Additionally, the potential for MPs to enter aquatic food webs through rivers, lakes, and the oceans fed by melting snow and ice highlights the need to explore the full range of ecological, chemical, and human health risks associated with this emerging contaminant.

The presence of MPs in the cryosphere also raises questions about their role in climate feedback mechanisms. MPs deposited on snow and ice may alter the albedo, increase the absorption of solar radiation, and potentially accelerate the rate of ice melt. This feedback mechanism could exacerbate global warming, with far-reaching consequences for freshwater availability, sea-level rise, and biodiversity. Furthermore, there is a lack of data on the effects of MPs on cryospheric ecosystems, including the potential for bioaccumulation and the disturbance of trophic interactions in these fragile environments. Further, challenges in standardizing detection methods, coupled with the logistical difficulties of sampling in extreme environments, hinder comprehensive assessments of MPs.

This review synthesizes current research on the occurrence, sources, and impacts of MP pollution in the cryosphere and builds upon the previous contribution,^17^ which provided a detailed overview of MP contamination across major cryospheric systems. Incorporating recently published evidence from high-altitude environments worldwide, including new observations from the Himalayan cryosphere, this review evaluates both atmospheric and hydrological pathways through which MPs infiltrate these remote regions and influence snow, ice, and meltwater systems, processes only partially addressed in earlier works. The review also assesses emerging modeling frameworks and monitoring techniques. Together, these advancements offer a more comprehensive and forward-looking understanding of MP contamination in the cryosphere.

## Characteristics of MPs in cryospheric environments

MPs detected in cryospheric environments exhibit diverse physical and chemical characteristics that provide insights into their sources, transport pathways, and environmental fate. Among these, the particle size, shape, polymer composition, and color are interlinked attributes that together determine the distribution of MPs and shape their ecological implications.

### Particle size distribution

**Figure 1:**
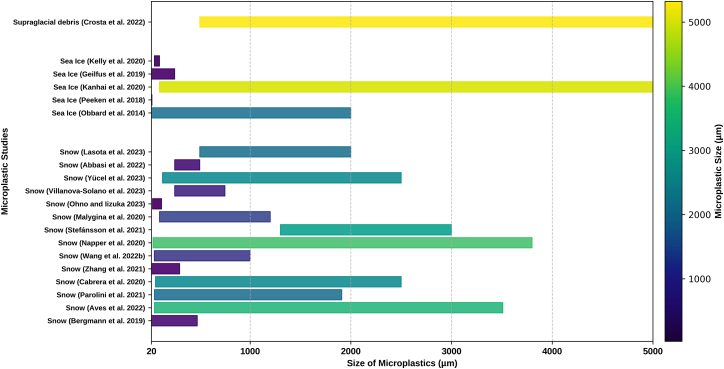
Comparative size analysis of MPs in snow, sea ice, and supraglacial debris across cryosphere regions

MPs reported in different cryosphere regions show significant variability in size (Figure 1) due to the varied sources^49^ and different filters and analytical methodologies used.^50^ Generally, it has been observed that MP abundance increases with decreasing size.^47^^,^^49^^,^^51^^,^^52^^,^^53^ Most cryosphere-related studies have observed MP sizes <50 μm (Table 1). In Arctic snow, 80% of MPs are ≤25 μm in size.^47^ In Laohugou No. 12 and Qiangyong glaciers, samples showed MPs with sizes less than <100 μm. Approximately 70% and 67% of MPs with sizes <11 μm were present in Arctic sea ice and snow, respectively.^47^^,^^54^^,^^55^ The prevalent particle size of MPs detected in the snow pits of the Tibetan Plateau (Demula glacier) is < 200 μm, with the smallest size of 36 μm in the glacier snow of Mount Everest.^56^ In the Arctic, 98% of MPs were <100 μm,^47^ while in the Antarctic snow, only 10% of MPs were <100 μm.^57^ However, larger MPs reported in the Antarctic region are due to local sources of pollution (textile fibers and other equipment from researchers). This contrasts with Arctic sea ice studies, where long-range transport mechanisms are believed to be the primary contributors to MP pollution.^39^^,^^43^

**Table 1:** Details of existing studies that have analyzed MPs from sea ice across the world

Sample type/study area	Sampling methods or models used	Method employed/Instrument used	Abundance/Size	Polymer Types/Shape/Color	Reference
Sea ice [Arctic Ocean]	The 1 m–3.5 m Sea ice cores were drilled, exterior sections were removed with razor blades, and then the interior sections were melted directly in precleaned glass bottles	**Filtration:** Nitrocellulose membranes (pore size 0.22 μm) **FTIR, Microscope**	**Abundance:** 38-234 MP L^−1^ **Size:** 20 - <2000 μm	**Type:** Rayon (54%), Polyester (21%), PA (16%), PP (3%), PS (2%), Acrylic (2%), PE (2%) **Shape:** Fibers, nodules, chips **Color:** Blue, red, green, black, orange	Obbard et al.^39^
Sea Ice [Arctic Ocean (Central Arctic)]	Snow was removed before drilling the sea ice cores. Nitrile gloves were used. Sea Ice cores were drilled with a Kovacs 9 cm diameter corer and were transported into plastic bags (polyethylene tube films) and stored at − 20°C. Ice cores were cut individually into horizons ranging from 10 to 35 cm using a bone saw.	**Filtration:** Aluminum oxide filters (pore size 0.2 μm) **Organic matter removal:** 35% H_2_O_2_ **Imaging FTIR (≥11 μm)**	**Abundance:** 1.1 × 10^3^ - 1.2 × 10^4^ MP L^−1^ **Size:** <25 μm (80%)	**Type:** PE (48%), PP (1.65%), EVA, CE alkylated, PES, PA, Varnish, Others (<1%)	Peeken et al.^54^
Sea Ice [Arctic]	Sea Ice cores were collected using a stainless-steel core barrel of 12.5 cm diameter, and then the ice cores were placed into clean bags (polyethylene) and transported to the laboratory. **Model:** AWI ICE Track application	**Filtration:** Glass microfibre filter (pore size 1.2 μm) **FTIR, Microscope**	**Abundance:** 2-17 MP L^−1^ **Size:** 100-5000 μm	**Type:** Polyester (57%), PA (19%), PU (6%), PAN (6%), Styrène/acrylates (6%), PVC (5%), other polymers (1.3%) **Shape:** Fibers **Color:** Red (10%), pink (9%), yellow (7%), black (5%), green (3.5%), transparent (3.5%), white (3%), gray (3%), orange, purple and brown (3%)	Kanhai et al.^58^
Free floating Sea Ice [Arctic (Svalbard)]	Sea ice was sampled directly using a metal boat book and a green plastic basket connected to a white rope, and was immediately placed in pre-rinsed (three times with filtered seawater, GF/F, 0.7 μm, Whatman) plastic buckets (white or	**Filtration:** Glass microfibre filter (pore size 0.7 μm) **FTIR, Stereomicroscope**	**Abundance:** 158 MP L^−1^ **Size:** >50 μm	**Type:** PET, PA, LDPE, PU, Paint, urethane alkyd Acrylic, Cellophane, Cotton, Wool	von Friesen et al.^37^
Sea Ice [Gulf of Bothnia of the Baltic Sea]	Sea ice cores were sampled using a ceramic knife and a Kovacs Mark II coring system (internal diameter 9 cm). Ice samples were wrapped in aluminum foil to avoid cross-contamination and kept frozen until further analysis.	**Filtration:** Glass microfibre filter (pore size 0.7 μm) **FTIR, Microscope**	**Abundance:** Upper layer of sea ice core: 758-39, 600 MP L^−1^ Below: 180–527 MP L^−1^ **Size:** <250 μm	–	Geilfus et al.^36^
Sea ice surface [Antarctica (Collins Glacier)]	Squares of ice surface (1 m^2^) were collected in a container pre-cleaned with Milli-Q water, wrapped in aluminum foil, and heated to 300°C for 4 h to remove organic matter.	**ATR-FTIR, μ-FTIR**	**Abundance:** EPS: 0.17–0.33 MP m^−2^ PET: 0.25 MP m^−2^ **Size:** 2292-12628 μm	–	González-Pleiter et al.^59^
Sea Ice [East Antarctica]	The sea ice cores were collected using a highly precise electro-polished stainless-steel core. The cores were triple-bagged in acid-cleaned plastic bags during transportation and stored in a −18°C freezer.	**Filtration:** Aluminum oxide filters (pore size 0.2 μm) **Organic matter removal:** 35% H_2_O_2_ **μ-FTIR**	**Abundance:** 11.71 MP L^−1^ **Size:** ≤50 μm (60%) <100 μm (90%)	**Type:** PE (34%), PP (15%), PA (14%), Varnish (11%), Resin (5%), EVA (4%), NBR (4%), PVA (4%), PS (3%), Silicone (2%), PES (2%), Rayon (1%)	Kelly et al.^60^
Sea Ice [East Antarctica, Ross Sea, Cape Evans]	Sea Ice core (Antarctica) Greenland firn core	**TD-PTR-MS**	**Abundance:** Greenland: 13.2 ng mL^−1^ Antarctica: 52.3 ng mL^−1^	**Type:** Greenland: PE (49%), PP, PET, PS, PVC, and tire wear NPs. Antarctica: PE (>50%), PP, PET	Materić et al.^61^

PU: polyurethane; PET: polyethylene terephthalate; PP: polypropylene; PE: polyethylene; PC: polycarbonate; PB: polybutadiene; PA: polyamide; PHR: phenoxy resin; CAB: cellulose acetate butyrate; CR: chloroprene rubber; PVC; polyvinyl chloride; PS: polystyrene; PES: polyester; LDPE: low-density polyethylene; PAN: polyacrylonitrile; EVA: ethylene-vinyl acetate; PMMA: polymethyl methacrylate; CN: cellulose nitrate; CE: cellulose acetate; PPS: polyphenol sulfone; ABS: acrylonitrile butadiene styrene; POLY: 2, 6-dimethyl-1 poly (2,6-dimethyl-1,4-phenylene oxide); HPC: hydroxypropyl cellulose; HPMC: hydroxypropyl methylcellulose; NBR: nitrile rubber; PPC: polypropylene carbonate.

The size of MPs has also been observed to fluctuate with seasonal variations. For instance, in the Demula Glacier, Tibet, smaller sizes (<200 μm) were more prevalent during the monsoon season, whereas in the non-monsoon season, MP with <500 μm were the predominant type.^55^ The influx of tourism also affects the quantity and characteristics of MPs,^45^ as there is an increase in both the presence and size of MPs following the tourist season.^62^

### Shape

Different shapes of MPs have been detected in cryosphere studies, including fibers, granules, films, and fragments. However, fibers are consistently reported as the dominant morphological type^17^^,^^50^^,^^55^^,^^56^^,^^63^^,^^64^^,^^65^ and are found across diverse regions such as the Arctic,^58^ Antarctic,^66^ Everest,^56^ Tibet,^51^ the Alps,^67^^,^^68^ and the Tropical Andes.^69^ This prevalence is attributed to their low density, distinct physical characteristics, and large surface area.^57^^,^^63^^,^^64^^,^^70^ Along a latitudinal gradient, airborne MP fragments decrease in concentration with increasing latitude (e.g., from mid-Northern Hemisphere to Antarctica), whereas fibers remain relatively constant due to their higher atmospheric transport efficiency.^71^

In the Forni Glacier, 65.2% of MPs detected were fibers,^68^ and in the Demula Glacier, fibers exceeding lengths >500 μm were dominant.^55^ Arctic snow contains fibers ranging from 65 to 14,314 μm, with 31% being shorter than 500 μm, while fibers in European snow are notably longer than those from Arctic snow.^47^ Fragments comprised 34.8% of Forni Glacier samples,^68^ whereas in Antarctic samples reported 39% of MPs as fragments with sizes <1000 μm, and 60% fibers spanning multiple size ranges.^57^

In the Ross Sea, Antarctica, fragments were predominant in seawater, comprising 72% MPs. In the snow cores of Vatnajökull Ice Cap, >90% of the MPs observed were fibers.^72^ Individual plastic fragments exhibit varying equal-area diameters, generally ranging from ∼30 to 3,000 μm.

In atmospheric samples, fibrous MPs accounted for 92% of MP deposition in London, UK, with deposition rates ranging from 575 to 1,008 MP m^−2^ day^−1^.^65^ In the Western Italian Alps, 39% of MPs detected were microfibers with lengths of 83-1,910 μm, and 61% were fragments with sizes of 50–422 μm long.^67^

Polyester fibers (94%) were also predominantly reported in the Mount Everest region.^56^ In the snow of Hokkaido, Japan, large-sized MPs were primarily fibers, while fragments were present in sizes <100 μm.^73^

### Polymer composition

**Figure 2:**
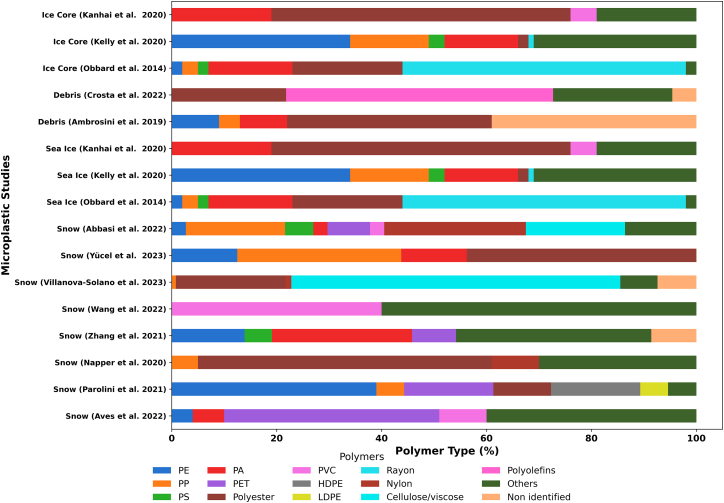
Comparative analysis of the polymer type of MP in snow, sea ice, and supraglacial debris across cryosphere regions PE: polyethylene, PP: polypropylene, PS: polystyrene, PA: polyamide, PET: polyethylene terephthalate, PVC: polyvinyl chloride, HDPE: high-density polyethylene, LDPE: low-density polyethylene.

The polymer composition of MPs in the cryosphere provides critical insights into their sources, transport pathways, and environmental persistence. There is significant variability in polymer composition reported in snow and ice (Figure 2). Diverse polymer compositions of MPs provide information about the origin and can tentatively help identify specific organic additives. However, polymer leachate composition shows variability across different polymer types.^74^^,^^75^

Studies have identified PE, PP, and PS as the most commonly detected polymers in cryospheric environments, reflecting their widespread use in packaging, textiles, and industrial applications.^47^^,^^76^ Rayon, PE, and PET dominated MP samples in Arctic sea ice, likely originating from fragmented plastic waste and synthetic fibers.^39^^,^^47^ In East Antarctica, 14 types of polymers have been identified, with PE, PP, and PA collectively accounting for 63% of the total MPs.^43^ Seventeen polymers were identified across Arctic ice cores.^54^ PET was detected in 79% of the Antarctic snow samples.^57^ In the Andes glacier samples, polyurethane (PU, 56%) was reported in high abundance.^69^

A high abundance of PET has also been observed in glaciers of the Austrian Alps,^76^ suggesting long-range atmospheric transport as a major pathway for MP deposition in remote cryospheric regions. Other studies have similarly found PET to be a dominant polymer in alpine winter surface snow and subglacial debris.^68^^,^^77^ On Mount Everest, the majority of MPs detected were polyester fibers.^56^ In Demula Glacier, PVC (40%) was reported as the dominant polymer,^55^ and has been attributed to sources such as automotive tire wear, the construction sector, and textile fibers. Subsequent studies have also detected PET, PA, PS, PE, and other polymers in dry and wet deposition samples collected from remote regions.^52^^,^^78^

In Antarctic sea ice, PE and PP have been reported in high abundance. PET has also been reported in a significant amount (87.23%),^79^ which contrasts with previous studies on Antarctic surface waters and sea ice.^43^^,^^80^ MPs undergo degradation from exposure to sunlight and mechanical forces. In this context, MPs detected in the snow samples appear more weathered and physically damaged, partly due to the contamination with extraneous geogenic particles.^49^

On Mount Everest, PET is the dominant polymer detected in MPs, followed by acrylic, nylon, and PP, likely originating from nearby clothing and equipment sources.^56^ PVC-dominated MPs have also been detected in the Demula Glacier snow pit.^55^ Polymer composition is further influenced by seasonal variations. For instance, PVC has been reported mainly during the non-monsoon season, which may be linked to its relatively higher density, limiting its potential for long-range atmospheric transport.

However, despite the increasing recognition of the presence of MPs in cryosphere regions, the potential impacts of these contaminants have not been thoroughly studied. In Antarctica, the presence of varnish and ethylene vinyl acetate has been attributed to the ship traffic around the continent, as these polymers are commonly used in vessel paints and fishing gear, and are largely composed of PE and PP.^43^ The presence of black rubber particles (PS, PBT, and nylon) has been linked to automobile traffic.^73^ Polyamide (PA), PP, and polyester have been associated with the clothing industry.^59^^,^^63^^,^^81^

In the studied mountain trails of Poland, the detection of PU has been attributed to the textile and footwear industries, travel bags, and sports gear.^45^

### Color

**Figure 3:**
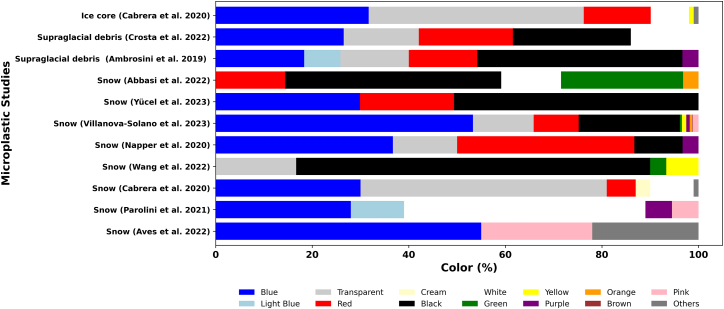
Comparative color analysis of MP across cryosphere regions

Different colors of MP have been reported in the snow and ice samples (Figure 3). These MPs exhibit a diverse range of colors, which significantly influence their environmental behavior, degradation pathways, and ecological impacts.^47^ Studies have shown that MPs in glacial, snow, and ice environments are frequently dominated by colors such as blue, red, black, and white, with variability largely attributed to source differences and weathering processes.^51^^,^^82^ However, blue and transparent particles are reported as the predominant colors in multiple studies.^67^^,^^77^^,^^83^

In the Southeast Tibetan Plateau (TP), black and transparent were reported as the dominant colors.^55^ In the Arctic Ocean, blue microplastics accounted for 53% of detected MPs, whereas in the Alps, white-colored MPs comprised 50% of detected particles.^67^ Blue and red MPs were the most prevalent colors reported in Arctic snow studies,^47^ with their presence tentatively linked to fragmented fishing gear and synthetic textiles. In Antarctic snow, blue MPs dominated both fragments and fibers (55%), followed by pink MPs (23%).^57^

## Source and pathways

**Figure 4:**
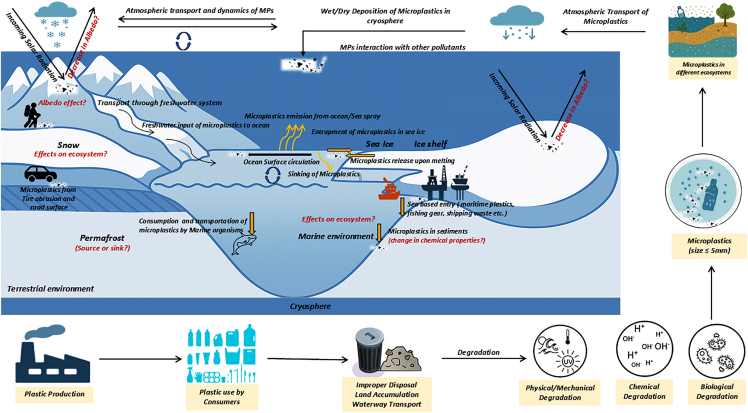
Schematic diagram shows the sources, pathways, and impacts of MPs in the cryosphere (modified after Vaughan et al.^86^) ∗Sea-based entry through maritime plastics, fishing gear, shipping waste, and so forth.

Microplastics are transported through atmospheric, riverine, and glacial processes^56^^,^^72^^,^^84^ (Figure 4). Among these, atmospheric transport has emerged as a dominant pathway, carrying MPs over vast distances from urban, industrial, and coastal regions into some of the most remote environments on Earth.^50^^,^^85^ Riverine and oceanic processes, along with local human activities, further contribute to MP accumulation, underscoring the combined influence of global and local sources.

### Atmospheric transport and snow deposition

Numerous studies have demonstrated the long-range transport potential of MPs, originating from diverse sources such as urban areas and industrial sites,^55^^,^^78^^,^^87^ with estimates of deposition fluxes varying by geographical location and seasonal influences.^88^ These particles travel vast distances, reaching high-altitude glaciers and polar regions.^43^^,^^51^^,^^58^ Long-range transport is facilitated by prevailing wind patterns and atmospheric circulation processes (e.g., wind direction and speed, vertical air movements, turbulence, and convective uplift). These processes efficiently carry MPs to both polar and non-polar high-altitude regions, where they subsequently settle on the snow and ice surface through both wet and dry deposition.^63^^,^^78^^,^^89^

In addition, tire and brake-wear particles from road traffic constitute a substantial fraction of atmospheric MP emissions.^90^ MPs entrapped within glacier ice as secondary materials may influence glacier melting and ice-flow characteristics, contributing to changes in local meltwater availability and potentially to the global rise in sea levels.^72^ However, MP size and type can vary depending on proximity to pollution sources and atmospheric transport conditions.^50^

Several factors also influence the accumulation rate of MPs in glacier systems, including altitude, which affects snow accumulation quality and melt rates, and proximity to pollution sources. Seasonal variations in atmospheric deposition and meltwater runoff influence the timing and spatial patterns of MP accumulation within the glacier. Glacial hydrology also plays a role, as meltwater can transport MPs from the surface into deeper ice layers, potentially leading to further accumulation within the ice mass.

Snowpacks act as significant sinks for atmospheric MPs, accumulating particles transported over long distances. Atmospheric transport and deposition are key processes governing MP abundance in snow. The efficiency of the atmospheric scavenging of MPs is influenced by several factors, including the particle size, shape, and density.^91^ Smaller and lighter MPs are more effectively transported over long distances and deposited in snowpacks.^50^ Latitudinal and altitudinal variations in atmospheric scavenging efficiency also affect MP deposition in snow, with higher deposition rates expected in areas closer to pollution sources and in regions with higher snowfall.^91^ Meteorological conditions, such as wind patterns and precipitation intensity, play a crucial role in determining the amount and type of MPs deposited on snow. Studies have reported varying MP concentrations in snowpacks across different geographical locations, highlighting the influence of local and regional pollution sources and atmospheric transport patterns.^56^^,^^57^ Nevertheless, further research is needed to improve understanding and to accurately quantify these processes and their regional variability.

### Sea ice and sea-to-air exchange

Sea ice, formed through the freezing of seawater, plays a critical role in the transport and storage of MPs in the polar regions.^92^ Several factors, including salinity, temperature, and ice dynamics,^93^ influence the concentration and characteristics of MPs entrapped in sea ice. Higher salinity can lead to greater MP incorporation during ice formation, while temperature variations affect the rate of ice growth and MP entrapment.^94^^,^^95^ Ice dynamics, including movement and melting, further regulate MP distribution within the ice and their eventual release into the marine environment. Studies have reported varying MP abundances and particle characteristics in sea ice across different regions, shaped by local and regional pollution sources and sea-ice formation processes.^54^

Once entrapped, sea ice acts as a temporary sink, storing MPs over the winter and releasing them during seasonal melt. During melting, these particles are released back into the water column, creating a seasonal redistribution mechanism that facilitates further oceanic transport.^92^ In addition to ice dynamics, this process constitutes a major pathway for long-range MP transport. Wave breaking, bubble bursting, and foam formation can lift MPs from surface seawater into the atmosphere, enabling their transport across hundreds to thousands of kilometers before deposition onto ice sheets or snowpacks.^96^ These mechanisms offer a plausible explanation for the presence of MPs in remote interior regions of the Antarctic and the Arctic, far from local anthropogenic sources, while complementing other atmospheric transport routes.

The combined action of sea ice dynamics, ocean currents, and sea-to-air exchange ensures that MPs are transported, trapped, and seasonally released, linking marine, atmospheric, and cryospheric compartments.^97^^,^^98^ In the polar oceans, sea ice serves as both a source and a temporary repository for MPs, influencing their spatial distribution and ecological impacts in high-latitude environments.

### Riverine and oceanic pathways

Aquatic transport through rivers and ocean currents also carries MPs to and within cryospheric environments.^91^^,^^99^ MPs originating from terrestrial sources are transported by river systems into the oceans,^88^ and are subsequently carried over vast distances by complex ocean currents.^91^ This process is particularly significant in regions with extensive river networks that drain into the polar oceans. For example, the Arctic Ocean receives considerable MP input from major rivers such as the Ob River, Yenisei River, and Lena River.^99^ These rivers transport substantial MP loads from their catchment areas, contributing to the overall MP burden in the Arctic Ocean.

Sea ice, a dynamic component of the cryosphere, serves as both a temporal sink and a source of MPs.^54^ During sea-ice formation, MPs present in the surrounding seawater are incorporated into the ice matrices. During ice melt, these MPs are released back into the water column, with potential impacts on the marine ecosystems.^54^ The interaction between sea-ice dynamics and MP distribution is influenced by various factors such as ice type, seasonality, and oceanographic processes.^60^ For instance, the formation of sea ice in the winter months can lead to higher MP concentration within the ice, while melting in the summer releases these particles back into the water column. The processes of ice formation and melt significantly influence the spatial distribution and concentration of MPs in the Arctic Ocean.

### Local human sources

Long-range transport dominates the distribution of MPs to the remote cryosphere regions. Local human activities, for example, tourism,^81^ synthetic-fiber clothing worn by expeditionists, ropes, tents, and flags, can also directly contribute to MP occurrence in these sensitive ecosystems.^56^ In the Ross Sea, Antarctica, local sources, including scientific research stations, field-based activities, sewage discharge, tourist influx, and fishing vessels, contribute to MP pollution.^40^^,^^100^ Likewise, in the Arctic, primary local sources associated with major maritime activities, including hydrocarbon exploration, aquaculture, shipping traffic, fisheries, and cruise tourism, add substantial MP loads.^91^ The influence of these local sources varies with the intensity of human activity and proximity to vulnerable ecosystems.^101^

Studies have reported higher MP concentrations in areas of increased human activity.^101^ For example, the discharge of wastewater from research stations and the improper disposal of fishing gear can lead to localized MP accumulation. Tourism-related maritime activities, particularly those involving cruise ships, also contribute to MP contamination in coastal regions.^101^^,^^102^ Antarctic coastal ecosystems similarly exhibit higher MP contamination levels compared to the open ocean.^103^ This localized increase has been linked to wastewater discharge from research and tourist vessels, alongside inputs from fishing-related activities. In contrast, the vast expanse of the Southern Ocean appears to show comparatively lower MP influence, indicating that the open ocean may act as a dilution zone for MPs originating from coastal sources.^104^^,^^105^ High-altitude cryospheric regions, such as the TP, also record considerable MP accumulation. The detection of MPs at such elevations >4400 m asl highlights the important role of long-range atmospheric transport in MP dispersal to remote and seemingly pristine environments. The mechanisms driving this atmospheric transport, such as prevailing wind patterns and broader atmospheric circulation processes, require further scientific investigation. MP accumulation in these high-altitude environments has substantial implications for both local ecosystems and global climate interactions.^55^

### Modeling approaches

Modelling studies^106^^,^^107^ provide valuable insights into the global atmospheric circulation patterns of MPs, refining the understanding of atmospheric residence times and deposition patterns.^52^^,^^78^ Several advanced modeling approaches can be employed to investigate the sources and pathways of atmospheric MPs. The Hybrid Single-Particle Lagrangian Integrated Trajectory (HYSPLIT) model, for example, analyzes historical air-parcel routes to trace the origins and movement of MPs at the global, regional, and local scales.^108^ By incorporating factors such as wind speed and precipitation, HYSPLIT provides a geographic context that enhances the understanding of atmospheric MP transport. The model can generate event-specific backward-trajectory maps and trajectory frequency fields, extending the investigations to distances of several thousand kilometres.^52^

In addition, the C-TRAIL model, combining a trajectory-grid Lagrangian advection method with a modified Eulerian approach, can simulate MP dispersion and accumulation, particularly in snow-covered regions.^109^ The TrajStat software, often used alongside HYSPLIT, provides a platform for visualizing air-mass pathways and supports trajectory clustering for source identification.^110^^,^^111^ By clustering trajectories and calculating metrics such as the Potential Source Contribution Function (PSCF) and Concentration-Weighted Trajectory (CWT), these modeling frameworks help pinpoint source regions contributing to MP pollution.^112^^,^^113^^,^^114^

Despite their advances, challenges remain in capturing the complex interactions between atmospheric variables and MP transport. Limitations related to model resolution, topographic representation, and the atmospheric data availability highlight the need for continued model refinement. These modeling systems integrate parameters such as particle size, wind speed, and precipitation to simulate MP movement and deposition across global cryospheric regions.

## Microplastic particles in polar latitudes and non-polar high altitudes

**Figure 5:**
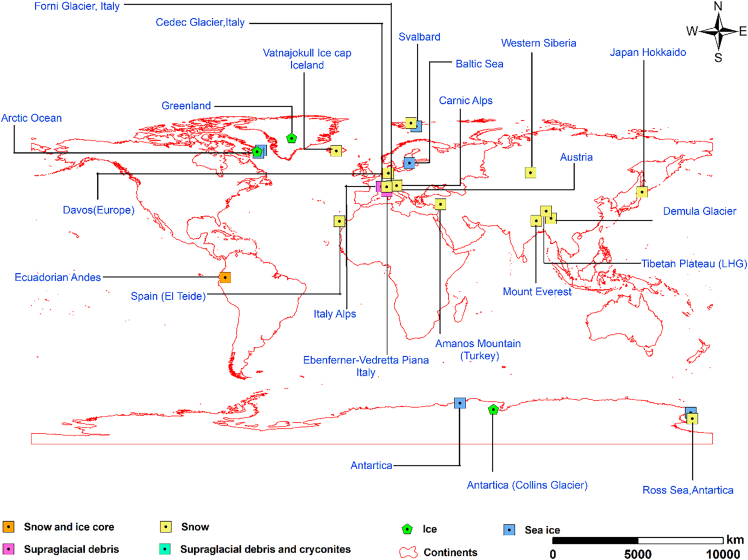
Global distribution of MP studies in cryospheric areas

MPs exhibit ubiquitous distribution across diverse cryosphere regions, including the Arctic, Antarctic, the Alps, the TP, and the Andes (Figure 5). However, the abundance, size distribution, shape, and polymer composition of MPs vary significantly across these regions.^50^ This heterogeneity reflects the complex interplay of sources, transport pathways, and environmental factors specific to each region, as well as differences in sampling and analytical methods used.

### Microplastic particles in the Arctic and Antarctic cryosphere

The Arctic cryosphere, encompassing sea ice, snow cover, the Greenland ice sheet, and permafrost, acts as a significant temporal sink and source for MPs. MP concentrations reported for different Arctic regions show substantial variability, spanning several orders of magnitude.^37^^,^^39^^,^^47^^,^^58^ These discrepancies are primarily attributed to differences in sampling techniques, analytical methods, and the inherent heterogeneity of cryospheric environments. Research conducted on Arctic sea ice has reported highly contrasting MP abundances, ranging from a few MP L^−1^ to several thousand.^36^^,^^37^^,^^39^^,^^54^^,^^58^ This variability underscores the complexity of MP deposition on sea ice and the influence of local-scale factors such as atmospheric inputs and sea spray contributions.

Arctic snow has also been identified as a reservoir for MPs, with patchy particle distributions reported in several studies (Table 2). For instance, elevated MP concentrations were measured in snow samples collected from ice floes traversing the Arctic Fram Strait and from various locations in the Svalbard Islands, with mean concentrations reaching 14.4 × 10^3^ MP L^−1^ and 1.1 × 10^3^ MP L^−1^, respectively.^115^ Overall, MP levels in Arctic snow ranged from 0 to 14.4 ×10^3^ MP L^−1^, with a reported mean of 1.76 ± 1.58 × 10^3^ MP L^−1^. These mean values were comparatively lower than those detected in snow from European sites (0.19×10^3^ - 154×10^3^ MP L^−1^).^47^ However, assessing the role of snow in MP deposition on sea ice remains methodologically challenging, partly due to difficulties in determining the environmental history of lying snow and redistribution prior to sampling. Recent research also suggests that sea spray may contribute to MP accumulation on sea ice, representing an important yet previously under-accounted source of MP contamination in these environments.

**Table 2:** Details of existing studies that have analyzed MPs from snow across the world

Sample type/study area	Sampling methods or models used	Method employed/Instrument used	Abundance/Size	Polymer Types/Shape/Color	References
Snow surface [Alps (Europe) and Arctic]	Surface snow was collected with a steel spoon, pre-rinsed mug, or soup ladle, and transferred into pre-rinsed glass jars	**Filtration:** Aluminum oxide filters (pore size 0.2 μm) **μ-FTIR (≥11 μm)**	**Abundance:** European snow: (0.191 × 10^3^-154 × 10^3^ MP L^−1^) Arctic snow: (0–14.4 × 103 MP L^−1^) **Size:** (11-475 μm), ≤25 μm (80%), <100 μm (98%)	**Type:** Rubber, PE, PA, PU, Acrylates, Varnish **Shape:** Fibers	Bergmann et al.^47^
Snow [Antarctica (Ross Island region)]	Snow samples were collected with a stainless steel scoop in 500 mL stainless-steel bottles with snow from the top 2 cm of the surface and kept chilled in freezer (−25°C) **Model:** HYSPLIT	**Filtration:** Whatman nitrocellulose membrane (pore size 0.45 μm) **Organic matter removal:** H_2_O_2_+Fenton’s reagent **Stereomicroscope, μ-FTIR**	**Abundance:** 29.4 ± 4.7 MP L^−1^ **Size:** ≤1000 μm (81%), 0–200 μm (28%), Mean size of fibers 850 μm, Mean size of fragments 200 μm	**Type:** PET (41%), Co-Polymers (17%), PMMA (9%), PVC (9%), PA (6%), PE (4%), Alkyd (4%), CN (4%), Other (6%) **Shape:** Fibers (61%), Fragments (39%), Films (1%) **Color:** Blue (55%), Pink (23%), Others (22%)	Aves et al.^57^
Snow [Italian Central-Western Alps, ∼2500 m asl]	Snow samples were collected using stainless steel spoons and then placed into 2 L glass jars, both of which were precleaned with acetone. The samples were transported to the lab and kept at 4°C in darkness during analysis.	**Filtration:** Cellulose filters (pore size 1 μm) **Density Separation:** NaCl (365 g L^−1^) **Organic matter removal:** 30% H_2_O_2_ **Stereomicroscope μ-FTIR**	**Abundance:** 2.32 ± 0.96 MP L^−1^ **Size:** 83-1910 μm	**Type:** PE (39%), PET (17%), PU (5.3%), HDPE (17%), PES (11%), LDPE (5.3%), PP (5.3%) **Shape:** Fibers (39%), Films (61%) **Color:** White (50%), Blue (28%), Light Blue (11%), Pink (5.5%), Purple (5.5%)	Parolini et al.^67^
Snow surface and snow pit [Alps (Austria), 3100 m asl]	Snow sampling was performed in 20 cm sections taken with a stainless-steel cylinder. Surface snow is placed in pre cleaned glass bottle.	**Filtration:** PTFE filter (pore size 0.2 μm) **TD-PTR-MS**	**Abundance:** 5.4–27.4 ng mL^−1^	**Type:** Snow pit: (PET≫), PVC, and PPC Surface Snow: PEE **Shape:** Fibers	Materić et al.^76^
Surface snow [Carnic Alps, 1872 m asl]	Surface snow was collected in precleaned glass bottles (750 mL) using bare hands and a precleaned steel spoon. Four replicates (3 L) were obtained from each site.	**Filtration:** Paper fiber filter disk (Pore size 6 μm) **Density Separation:** Oversaturated NaCl **Organic matter removal:** Crenon enzyme (37°C TRIS- buffered pH) **Stereomicroscope μ-FTIR**	**Abundance:** 0.11 MP L^−1^ **Size:** 75 × 220 μm	**Type:** PET **Shape:** Fragment **Color:** Light Blue	Pastorino et al.^77^
Surface snow and Ice core [Antisana Glacier, Ecuadorian Andes, 5753 m asl]	Surface snow samples were taken within 40 m × 40 m plots. To retrieve consistent snow volume (450 mL) a metal sampler was used, and samples were stored in 33 × 24 cm pre-cleaned plastic bags and stored in a cooler filled with dry ice to avoid melting.	**Filtration:** Borosilicate lab glass vacuum filtration equipment with Cellulose nitrate filters (pore size 0.45 μm) **Density Separation:** 20 mL NaCl (1200 kg m^−3^), 3000 rpm centrifugation for 5 min **Microscope (MPs >60 μm)**	**Abundance:** Ice core: 69.2 MP L^−1^ Snow surface: 101 MP L^−1^ **Size:** 60-2500 μm	**Shape:** In surface snow samples: Fibers (71.5%), Fragment (7.8%), others (20.7%) In ice core samples: Fibers (66.5%), Fragments (8.9%), others (24.7%) **Color:** In Surface snow: Transparent (51%), Blue (30%), White (9%), Red (6%), Cream (3%), other (1%) In Ice core: Transparent (45%), Blue (32%), Red (14%), White (8%), Yellow (1%)	Cabrera et al.^83^
Surface snow/Glacier [LHG glacier No.12, Qilian Mountains, Tibetan Plateau, 4460–5600 m asl]	Surface snow was collected in glass jars by using a steel spoon to prevent potential contamination	**Filtration:** Aluminum oxide filter (pore size 0.2 μm). Anodisc, Whatman **Density Separation:** Saturated NaCl (365 g L^−1^) **Organic matter removal:** 15% H_2_O_2_ **μ-FTIR Raman**	**Abundance:** ∼650–920 MPS L^−1^ **Size:** 25 μm (38%), 50 μm (80%)	**Type:** PA (26.7%), PE (13.9%), PET (8.4%), PS (5.2%), PC (3.4%), Rubber (9.7%), Polyacetal (6.5%), Varnish (3.3%), Unknown (8.6%)	Zhang et al.^51^
Snow pit [Demula Glacier, Southeast Tibetan Plateau, 5173 m asl]	A snow-pit with a depth of 1.65 m was collected from the accumulation zone with precleaned iron spoons and spatulas in 2 L glass bottles and kept frozen till samples are transported to the lab **Model:** HYSPLIT	**Filtration:** Polycarbonate Gold-Coated Membrane Filters (pore size 0.4 μm) **Microscope Raman Spectroscopy, SEM, EDS, Picarro isotope mass spectrometer**	**Abundance:** 9.55 ± 0.9 MP L^−1^ (Monsoon: 14.57 ± 0.99 MP L^−1^ Non-Monsoon: 8.03 ± 0.72 MP L^−1^) **Size:** >1000 μm (10%), 500–1000 μm (20%), 200-500 μm (26.67%), <200 μm (43.33%) Smallest size: 48 μm	**Type:** PVC (40%), POLY (23.33%), HPC (13.33%), HPMC (13.33%) **Shape:** Fibers (50%), Film (33.33%), Fragments (16.67%) **Color:** Black (73.33%), Transparent (16.67%), Yellow (6.67%), Green (3.33%)	Wang et al.^55^
Snow [Mount Everest, Southern slope, 5300-8440 m asl]	For the snow sample, stainless steel metal containers were used	**Filtration:** Whatman glass microfiber filter (pore size 1.6 μm) **FTIR microscope (size >30 μm)**	**Abundance:** 30 ± 11 MP L^−1^ **Size:** Detection (>30 μm) 36-3800 μm	**Type:** PES (56%), Acrylic (31%), Nylon (9%), PP (5%) **Shape:** Fibers (94.6%), Fragments (5.4%) **Color:** Red (36.7%), Blue (36.7%), Clear (13.3%), Black (10.0%), Purple (3.3%)	Napper et al.^56^
Snow cores [Vatnajokull Ice cap, Iceland, ∼1400 m asl]	Two snow cores (3 m long) were drilled by a PICO hand coring drill/auger and then carefully packed in aluminum foil and kept frozen until the lab.	**Filtration** **Optical microscopy,** **μ-Raman spectroscopy**	**Abundance:** 2-5 MPs per sample **Size:** Fibers: 1300–3000 μm Fragments: 30–500 μm	**Type:** PU, PVC, ABS, PA **Shape:** Fibers, Fragments	Stefánsson et al.^72^
Snow [Western Siberia (South)]	Snow samples were taken and then melted in the lab. at room temperature in glass containers sealed with foils. **Model:** HYSPLIT	**Filtration:** Fiberglass filters (pore size 0.2 μm) **Digital microscope, fluorescent dye (Nile Red), Wood lamp**	**Abundance:** 3 MPs per sample **Size:** Fibers, films: 1200 μm, Granules: 100–600 μm	**Shape:** Fibers, Granules, Films	Malygina et al.^85^
Snow [Hokkaido (Northern Island of Japan)]	Upper 5 cm was transferred with a stainless steel spoon from the snow deposited in a glass bottle (metal lid). Sampling was conducted with bare hands along the downwind	**Filtration:** Anodisc Whatman filter (pore size 0.2 μm) **Organic matter removal:** 30 mL of 30% H_2_O_2_ **Microscope, ATR-FTIR**	**Abundance:** 1.5 × 102–4.2 × 102 MP L^−1^ **Size:** Fragments (96%): 30–120 μm Fibers: >120 μm	**Type:** Alkyd, EVA, PE: 92%, rubber, PA, PU, PMMA, PVC, PAN **Shape:** Fragment (97%)	Zhang et al.^79^
Snow [El Teide National Park, Canary Islands, Spain, 2150-3200 m asl]	Snow was collected down to 5–10 cm depth using a metallic scoop in a 500 mL borosilicate glass lab. Flasks calcined at 550°C for 4 h	**Filtration:** Glass microfibre filter (pore size 0.45 μm) **Stereomicroscope μ-FTIR**	**Abundance:** Pristine area: 51 ± 72 MP L^−1^ Accessible areas: 167 ± 104 MP L^−1^ Climbing area: 188 ± 1 64 MP L^−1^ **Size:** Pristine area: 250–500 μm Accessible area: 500–750 μm	**Type:** Cellulosic (62.7%), PES (20.9%), Acrylic (6.3%), Nylon (1%), PP (0.8%), Azlon (0.8%), Nonidentified (7.4%) **Shape:** Fibers (99.4%), Fragment (0.2%), Tangled Messes (0.2%), Films (0.2%) **Color:** Blue (52.6%), Black (20.7%) Translucent (12.4%), Red (9.2%), Pink (1.2%), Fluorescent Yellow (0.9%), Gray (0.9%), Purple (0.7%), Green (0.4%), Yellow (0.4%), Orange (0.5%), Brown (0.1%)	Villanova-Solano et al.^42^
Snow [Amanos Mountain, Hatay region, Turkey, 1500-2000 m asl]	1 m2 area was selected and a 5 cm thick snow sample was taken with metal spoons and transferred to metal buckets. **Model:** HYSPLIT	**Filtration:** Filters (pore size 50 μm) **Microscope ATR-FTIR**	**Abundance:** 10.5–16 MP L^−1^ (519 MPs in snow samples) **Size:** Fibers: 500–2500 μm Fragments: 130–380 μm	**Type:** PS (35%), PP (25%), PE (10%), PA (10%) **Shape:** Fiber (99%) **Color:** Black (47.6%), Blue (28.1%), Red (18.3%)	Yücel^38^
Fresh snow [Northern Iran, 1350 m asl]	Snow was collected in 2 L glass jar pre cleaned with distilled water using stainless steel spoon upwind, wrapped in aluminum foil and transferred to lab. **Model:** HYSPLIT	**Filtration:** Johnson cellulose test membranes (pore size 1 μm) **Density Separation:** ZnCl_2_ **Organic matter removal:** 30% H_2_O_2_ **SEM-EDX, μ-Raman spectroscopy**	**Abundance:** 0-86 MP L^−1^ **Size:** Urban snow: 250–500 μm Remote Snow: <100 μm	**Type:** Nylon (27%), Cellulose (18.9%), PP (18.9%), PS (5.4%), PET (8.1%), PVC (2.7%), PE (2.7%), PU (2.7%), PA (2.7%), Silicone (2.7%), Silk (2.7%), PPS (5.4%) **Shape:** Fibers (89.6%), Films (3.2%), Fragments (2.6%), Spherules (4.6%) **Color:** White Transparent (12.4%), Yellow Orange (3.2%), Red Pink (14.4%), Black Gray (44.8%), Blue Green (25.3%)	Abbasi et al.^49^
Snow [Trails to Babia Gora, Koscieliska valley, Izerska Meadow (Poland)]	With the help of a metal sampler, five samples of snow with a 1 L volume were taken to the lab for analysis	**Filtration:** Glass microfibre filters (pore size 0.45 μm) **FTIR microscope**	**Abundance:** Trails: Babia Gora: 5.2 MP L^−1^ Koscieliska valley: 12.9 MP L^−1^ Izerska Meadow: 9.8 MP L^−1^ **Size:** <500 μm (>50%), 600-1000 μm, 1100-2000 μm	**Type:** PU, PET, PP, PE, PC, PA, PHR, PB, CAB, CR **Shape:** Fragments, Fiber, Fiber balls, flakes **Color:** Black, White, Gray, Green, Red, Blue, Pink, Yellow	Lasota et al.^45^
Snow [Northeastern China]	The surface layer of seasonal snow (top 5 cm) was collected using a stainless-steel spoon transferred to a 2 L jar, and taken to the lab for analysis. **Model: HYSPLIT**	**Filtration:** Aluminum oxide filters (pore size: 0.22 μm) **μ-FTIR, Fluorescent microscope**	**Abundance:** (4.52 ± 3.05) ×10^4^ MP L^−1^ **Size:** 20–3500 μm **<**100 μm (>85%) <50 μm (69.4% ± 15.0%) 50–100 μm (14.0% ± 7.2%)	**Type:** EVA, PE, PA, PET, cellulose, cellophane **Shape:** Fragments, films, foams, fibers	Wen et al.^48^

PU: polyurethane; PET: polyethylene terephthalate; PP: polypropylene; PE: polyethylene; PC: polycarbonate; PB: polybutadiene; PA: polyamide; PHR: phenoxy resin; CAB: cellulose acetate butyrate; CR: chloroprene rubber; PVC; polyvinyl chloride; PS: polystyrene; PES: polyester; LDPE: low-density polyethylene; PAN: polyacrylonitrile; EVA: ethylene-vinyl acetate; PMMA: polymethyl methacrylate; CN: cellulose nitrate; CE: cellulose acetate; PPS: polyphenol sulfone; ABS: acrylonitrile butadiene styrene; POLY: 2, 6-dimethyl-1 poly (2,6-dimethyl-1,4-phenylene oxide); HPC: hydroxypropyl cellulose; HPMC: hydroxypropyl methylcellulose; NBR: nitrile rubber; PPC: polypropylene carbonate.

Antarctica, the southernmost continent on Earth, is dominated by a massive ice sheet with thicknesses of up to 4,500 m, accounting for approximately 90% of the freshwater reserves of the planet.^116^ Despite minimal direct human activity, MPs have been detected in Antarctic snow, ice, and surface waters, demonstrating the far-reaching influence of global plastic pollution.^117^ MP concentrations reported for East Antarctic sea ice^43^ (11.7 MP L^−1^) are higher than those in Antarctic surface waters (3.1 × 10^−5^ MP L^−1^) and continental coastlines (9.9 × 10^−5^ MP L^−1^), although substantially lower than the wide range reported for Arctic sea ice (33–75,413 MP L^−1^). Interestingly, MPs detected in East Antarctic ice^43^ (11.71 MP L^−1^) are higher than those in Antarctic surface waters,^105^ suggesting that sea ice may represent an important accumulation and transport reservoir for MPs. These findings indicate that Antarctic sea ice, such as Arctic sea ice, can act as a temporary reservoir for MPs, with deposition shaped by long-range atmospheric transport, oceanic currents, and local-scale cryosphere-ocean interactions.

Collectively, the polar regions act as both sinks and transient sources for MPs. Although concentrations reported here are generally lower than those measured close to human-activity hotspots, the presence of MPs in these remote environments underscores the global scale of plastic pollution and highlights the importance of long-range atmospheric transport and cryospheric processes in shaping MP distribution.

### Microplastic particles in the Tibetan Plateau and other alpine regions

The TP contains vast areas at an average altitude exceeding 4,000 m and hosts the largest glacier reservoirs outside the polar regions.^118^ Despite its remote location, MPs have been reported in multiple environmental compartments, including rivers, lakes, soils, glaciers, and the atmosphere.^119^ Investigations on glaciers such as northern Laohugou Glacier, southern Demula Glacier, and Mount Everest have reported abundances of 650–920 MP L^−1^, 9.6 ± 0.9 MP L^−1^, and 17 ± 14 MP L^−1^, respectively.^56^^,^^120^^,^^121^ These findings highlight substantial spatial variability among glacier systems and indicate that glacial snow and ice act as both temporary sinks and potential secondary sources for MPs during melt phases.

Investigations of MPs in the TP reveal moderate-to-low contamination compared to other cryospheric regions worldwide. In the Demula Glacier snow pit, the average MP abundance was 9.55 ± 0.9 MP L^−1^, dominated by fibers and films smaller than 200 μm.^55^ Comparative studies indicate that MP concentrations in the TP are broadly similar to those observed on the south slope of Mount Everest (17 ± 14 MP L^−1^), yet approximately one order of magnitude lower than abundances reported from the Antisana Glacier in the Andes.^56^^,^^83^ In contrast, substantially higher MP abundances (650–920 MP L^−1^) have been reported from the northern Laohugou Glacier, which underscores the strong spatial heterogeneity of MP deposition within the TP.^121^

When placed in a global context, MP concentrations in the TP and other high-altitude regions are lower than the extreme values recorded in parts of Europe. The lowest MP concentration worldwide has been reported from the Carnic Alps (0.11 MP L^−1^).^77^ However, exceptionally high levels (0.19 × 10^3^–154 × 10^3^ MP L^−1^)^47^ have been reported in Northern European snow, particularly from regions near Bremen and Bavaria.^47^^,^^77^ Such contrasts are largely attributed to methodological differences in polymer identification and particle-classification techniques, as well as the intensity of anthropogenic activities close to sampling sites.^55^^,^^58^

Alpine glaciers outside the TP provide further evidence of MP accumulation in remote mountain environments. In the Forni Glacier (Italian Alps), MPs have been detected in cryoconite holes and supraglacial debris, with a reported average abundance of 74.4 ± 28.3 MP kg^−1^ of sediment (dry weight), comparable to contamination levels observed in European coastal and marine sediments (Table 3).^68^ Similarly, studies in the Swiss Alps have revealed notable MP contamination in surface snow, with altitude and tourism identified as key influencing factors.^56^ Collectively, these findings demonstrate that the TP and other alpine regions, despite their remoteness, are not pristine. They function as temporary sinks for MPs transported via long-range atmospheric transport pathways and subsequently emerge as secondary sources through meltwater export into downstream aquatic ecosystems. This dual role underscores the importance of mountain glaciers as both receptors of global pollution and critical contributors to MP redistribution and ecosystem contamination beyond the cryosphere.

**Table 3:** Details of existing studies that have analyzed MPs from supraglacial debris across the world

Sample type/study area	Sampling methods or models used	Method employed/Instrument used	Abundance/Size	Polymer Types/Shape/Color	Reference
Supraglacial debris and cryoconite samples [Forni glacier (Italian Alps), 2580 m asl]	Samples are collected in glass jars with a metal shovel or a metal lab spoon, precleaned with acetone	**Filtration:** Glass fiber filters (pore size 0.45 μm) **Density Separation:** NaCl (365 g L^−1^) **Organic matter removal:** 15% H_2_O_2_ **FTIR Microscope**	**Abundance:** 74.4 ± 28.3 MP kg^−1^ dw	**Type:** PES (39%), PE (9%), PP (4%), PA (9%), Unknown (39%) **Shape:** Fibers (65.2%), Fragments (34.8%) **Color:** Black (51%), Blue (22%), Red (17%), Transparent (17%), Light Blue (9%), Violet (4%)	Ambrosini et al.^68^ 2019
Supraglacial debris [Central Alps, Northern Italy (Forni, Cedec, and Ebenferner-Vedretta Piana glaciers)]	Supraglacial debris samples were collected using a precleaned metal spoon and transferred to glass jars topped with aluminum foil.	**Filtration:** Cellulose filters (pore size 1 μm) **Density Separation:** NaCl **Organic matter removal:** 30% H_2_O_2_ **μ-FTIR**	**Abundance:** Forni glacier: 0.033 ± 0.007 MP g^−1^ dw Cedec glacier: 0.025 ± 0.009 MP g^−1^ dw Ebenferner-Vedretta Piana glacier: 0.265 ± 0.027 MP g^−1^ dw **Size:** 500-5323 μm	**Type:** Polyolefins (50.9%), polyesters (21.8%), others (12.4%), and acrylates (10.3%), unknown (4.7%) **Shape:** Fragment (71.4%), Fibers (28.6%) **Color:** Blue (22.7%), Black (20.9%) Red (16.7%), Transparent (13.3%), White (12%), Gray (9%)	Crosta et al.^41^ 2022

PU: polyurethane; PET: polyethylene terephthalate; PP: polypropylene; PE: polyethylene; PC: polycarbonate; PB: polybutadiene; PA: polyamide; PHR: phenoxy resin; CAB: cellulose acetate butyrate; CR: chloroprene rubber; PVC; polyvinyl chloride; PS: polystyrene; PES: polyester; LDPE: low-density polyethylene; PAN: polyacrylonitrile; EVA: ethylene-vinyl acetate; PMMA: polymethyl methacrylate; CN: cellulose nitrate; CE: cellulose acetate; PPS: polyphenol sulfone; ABS: acrylonitrile butadiene styrene; POLY: 2, 6-dimethyl-1 poly (2,6-dimethyl-1,4-phenylene oxide); HPC: hydroxypropyl cellulose; HPMC: hydroxypropyl methylcellulose; NBR: nitrile rubber; PPC: polypropylene carbonate.

### Seasonal variations

Seasonal dynamics strongly influence the distribution and characteristics of MPs in cryospheric environments. During the monsoon season, smaller particles (<200 μm) dominate, likely due to enhanced fragmentation and hydrological inputs, whereas relatively larger MPs (<500 μm) are more common during the non-monsoon season.^55^ Seasonal differences in MP colors have also been observed, with black MPs predominating during the monsoon, while a diverse color has been found in the non-monsoon period.^122^ This may be attributed to the influence of intense rainfall and surface runoff during the monsoon, which enhances plastic fragmentation and hydrological transport, leading to a dominance of smaller and darker MPs largely sourced from road-runoff pathways. In contrast, reduced hydrological disturbance during the non-monsoon period allows the accumulation of relatively larger and more variably colored MPs from multiple terrestrial and atmospheric sources.

Polymer composition shows comparable seasonal variability. For example, PVC concentrations are reported to be higher in the non-monsoon period, although MP abundance remains greater during the monsoon (14.57 ± 0.99 MP L^−1^ versus 8.03 ± 0.72 MP L^−1^).^55^ Cryospheric processes, such as seasonal freeze-thaw cycles, further contribute to the physical fragmentation of larger plastics and enable vertical redistribution of MPs within ice layers.^96^ For instance, sea ice in East Antarctica exhibited strong vertical heterogeneity, with MP concentrations of 31.6 MP L^−1^ at the bottom layer, 22.4 MP L^−1^ at the surface layer, and 0.71 MP L^−1^ in the middle layer.^43^ Collectively, these observations indicate that MP size, color, polymer type, and vertical distribution are strongly governed by seasonal climatic forcings and cryospheric processes.

### Microplastic particles in the Himalayan cryosphere

The Himalayan mountainous landscapes are ecologically diverse and provide essential services to inhabitants, but are also vulnerable to various stressors, including climate change, aerosols, and MP pollution. Several factors contribute to the presence of MPs in the Himalayan region, including population density, the distribution of MPs through rainfall and their transport via winds, river discharges, and improper plastic waste disposal.^123^ These processes may affect both the health of the local populations and aquatic ecosystems.

Recent research on high-mountain lakes has identified anthropogenic activities, particularly from the automobile, textile, and packaging industries, as key potential sources of MPs.^124^ Due to the lightweight nature of MPs, these small particles are transported through atmospheric processes and can disperse across vast areas of the Himalayan landscapes.

The ongoing retreat of glaciers, which act as repositories of accumulated MPs, may release embedded MPs into downstream water systems, enabling further transfer into natural and urban aquatic reservoirs. The Himalayan region possesses the second-largest ice mass globally after the polar regions, and serves as a major source for numerous major river systems. Different studies have reported the presence of MPs in rivers,^125^^,^^126^^,^^127^^,^^128^^,^^129^ lakes,^124^^,^^130^^,^^131^^,^^132^ and glaciers,^133^ collectively, demonstrating the significant extent of MP pollution in the Himalayan region. Despite this, research on MP occurrence, spatial fluxes, and transport pathways in glacial environments remains limited compared to sediment, coastal, riverine, and lacustrine environments. A detailed investigation of MP-transport dynamics of MP transport is essential to improve the understanding of MP deposition, dispersion, and accumulation mechanisms in Himalayan glacier systems, their seasonal release during snow and glacier melt, and their impacts on mountain ecosystems and associated biota.

As a first step in this direction, the HYSPLIT model^134^ has been used to quantify the global, regional, and local air mass trajectories reaching the glaciers in the Kashmir Valley, located in the Greater Himalayan mountains. The model has been extensively tested for tracking pollutant and moisture sources across diverse mountain regions worldwide.^59^^,^^135^^,^^136^^,^^137^ In this study, 168-h (7-day) backward trajectories were computed for each observation day at 14:00 h Indian Standard Time (IST), initialized at 500 m above the ground level. The analysis covers the period from December 2021 to November 2022. For trajectory generation, the HYSPLIT model used weekly meteorological inputs from the Global Data Assimilation System (GDAS) at 1 ° × 1 ° spatial resolution. These meteorological data were sourced online from the National Center for Environmental Prediction (NCEP), USA. Trajectory outputs were downloaded as shapefiles from the HYSPLIT-WEB interface and further analyzed on a seasonal basis (December-February: winter; March-May: spring; June-August: summer; and September-November: autumn) and by proximity to glacier regions. Trajectories originating from the Kashmir region were classified as local sources. The trajectories from the neighboring areas as regional sources, and long-distance trajectories as global sources.

**Figure 6:**
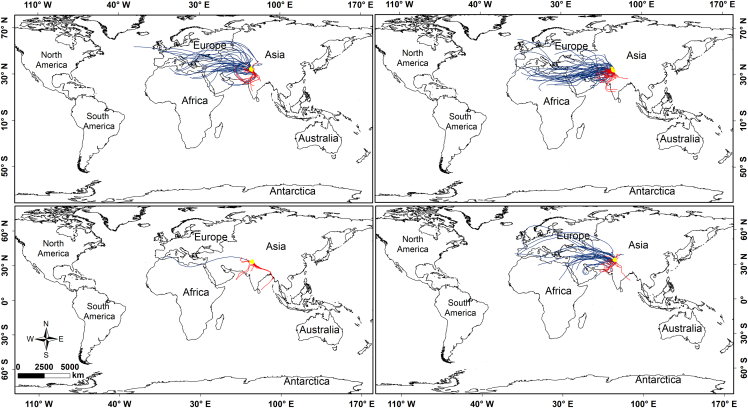
HYSPLIT back trajectory analysis of seasonal air masses reaching the glaciers over the Kashmir valley in the Greater Himalayan mountains (A) winter, (B) spring, (C) summer, and (D) autumn.

The 168-h backward trajectories from December 2021 to November 2022 show that out of a total of 365 trajectories, 156 (43%) originate from global sources via westerlies from the Mediterranean, 135 (37%) originate from regional sources, and 74 (20%) originate from local sources (Figure 6). In the winter season (Figure 6A), most air masses originate from global sources (52 trajectories, 58%), followed by the regional sources (33 trajectories, 37%), with a smaller proportion from the local sources (5 trajectories, 5%). In spring (Figure 6B), 59 trajectories (64%) originate from global sources, 30 trajectories (33%) originate from regional sources, and 3 trajectories (3%) originate from local sources. In summer (Figure 6C), the regional sources have the highest contribution (51 trajectories, 56%), while global influences are relatively lower (5 trajectories, 5%). During autumn (Figure 6D), 40 trajectories (44%) originate from global sources, 30 trajectories (33%) originate from local sources, and 21 trajectories (23%) originate from the regional sources. These findings highlight the need to undertake comprehensive modelling-based investigations to better understand MP sources over the high-mountain environments, including the Himalayan region, of South Asia.

## Interactions of microplastic particles in the cryosphere

Although the presence of MPs in snow, ice cores, and glacial meltwater has been documented, the broader interactions of MPs within cryospheric systems remain poorly understood.^96^ The cryosphere comprises multiple components, including snow, glaciers, permafrost, and seasonally frozen ground, each with distinct physical and chemical properties that can influence the transport, environmental fate, and accumulation dynamics of MPs.^138^^,^^139^

Once deposited via atmospheric transport or from localized anthropogenic activities, MPs may undergo cryogenic-weathering processes,^140^ such as mechanical fragmentation,^141^ UV degradation,^142^ and repeated freeze-thaw cycles.^143^ These mechanisms contribute to the breakdown of large particles into small fragments or nanoparticles, which exhibit enhanced mobility, reactivity, and potential toxicity.^144^^,^^145^ In snow and firn, MPs may also migrate vertically through meltwater percolation,^146^ leading to their long-term sequestration in glacier ice.^147^ This process can delay the release of MPs into downstream environments by several decades.^148^

A particularly underexplored aspect of MP interactions in the cryosphere is their behavior in permafrost environments.^66^ With the accelerating thaw under global warming, historically trapped MPs in frozen ground may re-enter active hydrological and ecological systems.^149^ MPs may act as sinks for hydrophobic contaminants, including PAHs,^150^ polychlorinated biphenyls (PCBs),^151^ and heavy metals,^152^ which may desorb because of temperature, pH, and salinity changes.^32^^,^^153^ This remobilization reintroduces MPs both as physical pollutants and vectors for chemically bound toxic substances, posing a dual contamination risk.^154^^,^^155^ Such synergistic threats in glacial and proglacial systems can cascade into alpine aquatic ecosystems and downstream communities, especially populations dependent on glacier-fed water sources.^156^

In seasonally frozen soils and snowpacks, freeze-thaw cycles further modulate MP mobility.^157^ During thaw periods, MPs may be released into supraglacial and proglacial meltwater streams, while refreezing events may temporarily immobilize the particles, creating intermittent contaminant fluxes.^147^ Moreover, glacial hydrology, comprising preferential flow paths such as moulins, crevasses, and subglacial channels, may facilitate long-distance transport of MPs within ice bodies, ultimately discharging them into proglacial lakes and the rivers.^158^

These interactions suggest that the cryosphere is not merely a passive sink but an active and dynamic reservoir that modulates the storage, transformation, and transport of MPs. As global warming accelerates cryospheric degradation, including the melting of snow and glacier ice and the thawing of permafrost, there is an increased potential for the remobilization of previously sequestered MPs and their associated contaminants. This accelerated release may alter the contaminant dynamics within glacial and proglacial systems, posing risks to alpine freshwater ecosystems and downstream human communities. Addressing these challenges requires coordinated interdisciplinary efforts that integrate high-resolution field observations, remote sensing, controlled laboratory experiments, and process-based modeling to elucidate the fluxes, fate, and ecological and biological impacts of MPs in cold-region environments.

## Impact of microplastic particles on cryosphere environments

The melting and shrinkage of ice in the cryospheric regions due to global warming has resulted in permafrost degradation, a decrease in sea-ice extent, and accelerated melting of glaciers and ice sheets^159^^,^^160^^,^^161^^,^^162^^,^^163^^,^^164^^,^^165^ (Figure 1). This process has the potential to disrupt the hydrological cycle and affect downstream areas.^166^^,^^167^ Climate change is also projected to significantly alter the dynamics of MP pollution in the cryosphere.^168^^,^^169^ Rising global temperatures will accelerate glacier, ice-sheet, and sea ice melt, leading to the release of previously stored MPs into the surrounding environment.^168^ The magnitude of this release depends on MP concentrations within ice and on melt rates, both of which are climate-sensitive. This creates a positive feedback loop in which climate change accelerates release of MPs, while increasing MP abundance on snow and ice surfaces may further enhance warming through processes such as albedo reduction and increased solar-radiation absorption.

Previous studies have demonstrated that dark-colored substances (such as black carbon and dust) significantly reduce surface albedo and promote melting.^170^^,^^171^ Similarly, different-colored MPs act as heat-absorbing particulates,^172^ exacerbating melt rates by increasing solar-radiation absorption.^47^ This effect creates a positive feedback loop, whereby MP presence amplifies climate-change impacts on the cryosphere. For example, in Arctic sea ice, MPs may increase sea-ice permeability and enhance solar radiation absorption, reducing surface albedo by ∼11% and contributing to sea-ice melt.^54^^,^^173^ In addition, recent studies report that airborne MPs exert an effective radiative forcing (ERF) of 0.044 ± 0.399 mW m^−2^ assuming a uniform distribution up to 10 km altitude, and −0.746 ± 0.553 mW m^−2^ when limited to the boundary layer. Although these values remain small relative to the total aerosol ERF (−0.71 to −0.14 mW m^−2^), continuing increases in plastic production indicate that atmospheric radiative effects of MPs may grow in the future.^172^ Furthermore, MPs may affect climate dynamics by acting as cloud ice nuclei^174^ and contribute incrementally to the global radiative forcing.^172^

Beyond their role in cryosphere-climate feedbacks, MPs also act as vectors for POPs, whereby they adsorb organic contaminants that may bioaccumulate in aquatic systems when melting-induced MP release transfers bound chemicals into downstream water bodies.^69^ MP-POP interaction occurs through hydrophobic interactions, electrostatic interactions, and other non-covalent forces (e.g., halogen bonding, π-πinteractions, and hydrogen bonding).^175^ The adsorption process is controlled by several factors, including the polarity of MPs, their aging degree, crystallinity, specific surface area, and particle size, as well as environmental parameters such as pH, temperature, and ionic strength. Pollutant-specific properties, such as hydrophobicity and dissociated chemical forms, further regulate sorption behavior.^175^ Experimental evidence demonstrates that perfluorooctanesulfonate (PFOS) sorbs efficiently onto PE microplastics. Smaller PE particles consistently adsorb higher PFOS concentrations, and 70–80% of PFOS can desorb under simulated fish gut conditions, indicating a potential trophic transfer and chemical transport across food chains.^176^

The presence of MPs in remote ice and snow deposits also threatens fragile ecosystems. These particles can be ingested by microbial and invertebrate communities, potentially affecting trophic interactions and introducing toxic chemicals into food webs. For example, Antarctic amphipods were observed ingesting 20–45 MPs per individual over a 48-h exposure period, demonstrating the potential for bioaccumulation.^177^ Increased MP ingestion, entanglement, and bioaccumulation represent major concerns.^91^ The pervasive nature of plastic pollution in the Arctic also affects terrestrial and aquatic systems.^91^ The ingestion of MP in juvenile polar cod, a keystone species in the Arctic food webs, illustrating the potential for trophic transfer, has also been documented.^178^ MPs have also been reported on Arctic beaches, suggesting potential pathways for ingestion by various organisms.^101^ The consequences of MP ingestion may vary depending on the organism, particle size, and polymer type. Physical damage, blockage of digestive tracts, reduced feeding efficiency, and impaired nutrient uptake are common effects.^91^^,^^178^ The potential bioaccumulation of MPs and their associated contaminants poses a further threat, particularly for apex predators.^179^

The cumulative effects of MP and other stressors, such as ocean acidification and warming temperatures, are likely to exacerbate the negative impacts on ecosystem resilience. MP-distribution hotspots have also been mapped in the Southern Ocean, considering interactions with climate change stressors.^180^ Seasonal entrapment and melt-driven MP release ensure extended bioavailability, increasing risks of ingestion by marine organisms.^181^ Recent studies also report that some marine species can fragment ingested MPs into nanoplastics during digestion,^182^ highlighting continued nano-remobilization potential in Antarctic marine ecosystems.^43^ Several studies have also detected MP in various Antarctic marine biota and environmental matrices, including sediments, benthic organisms, krill, and fish.^104^ In particular, Antarctic krill (Euphausia superba) were found to contain 0.29 ± 0.14 MPs per individual in the South Shetland Islands and 0.20 ± 0.083 MPs per individual in the South Orkney Islands.^181^ More than 90% of the detected MPs were <150 μm, >90% were fibers, with polymers such as PE (37%), PP (22%), and PET (21%). However, confirmed evidence of MP transfer to higher trophic levels in the Antarctic cryosphere, such as seabirds and marine mammals, remains limited.^104^

Cryospheric invertebrates, such as crustaceans (e.g., amphipods and copepods), insects (e.g., chironomids), and other benthic organisms, are crucial components of cryospheric food webs.^183^ MPs can accumulate in their tissues through ingestion,^184^ direct contact,^104^ or environmental uptake from the surrounding matrices.^185^ Exposure to MPs can induce oxidative stress,^186^ resulting to cellular damage^187^ and impaired physiological function.^188^ MP-associated inflammation can disrupt normal physiological processes and may cause progressive tissue damage.^189^ Reproductive toxicity, potentially affecting fertility and reproductive success, is another potential consequence of MP exposure in cryospheric invertebrates.^190^ The relatively low metabolic rates of many cryospheric invertebrates may influence their sensitivity to micro- and nano-plastic (MNP) toxicity. However, the effects of MPs on the cryospheric organisms remain largely unknown, and current evidence is insufficient to comprehensively assess ecological and organism-level consequences.

Fish inhabiting cryosphere environments, such as Arctic char, icefish, and other cold-water species, are important components of cold-region food webs and a crucial food source for humans.^191^ MNPs can accumulate in fish tissues through the ingestion of contaminated prey or by direct uptake from the surrounding water.^192^ Exposure to MNPs may result in multiple adverse effects, including growth inhibition, reproductive dysfunction, and behavioral changes.^193^ Trophic transfer of MNPs across food webs, from the lower trophic levels to fish, and the potential for biomagnification^194^ require further scientific investigation. The accumulation of MNPs in fish tissues may also pose risks to human health through the consumption of contaminated seafood^195^

Although research on the impacts of MPs in the cryosphere is still in its infancy, there is a need to understand the long-term fate and ecological impacts of MPs in the cryosphere.^88^^,^^104^ Future studies need to investigate the interactions of MP with other pollutants, organisms, and the carbon cycle, including the potential effects on ecosystem functioning. The role of biofouling, the process by which organisms colonize MP surfaces, requires further exploration as it can alter the physical and chemical properties of MPs and influence their transport, environmental fate, and ecological impacts. In addition, the potential for MP degradation in the cryospheric environments and the ecosystem-level consequences of this degradation remain important under-researched areas. These emerging concerns collectively emphasize the need to better understand the long-term consequences of MP pollution for cryospheric biodiversity, trophic interactions, and ecosystem functioning.

## Prospects

The presence of MP in the cryosphere poses several concerning environmental and climatic impacts. Over time, MPs can act as sinks for atmospheric pollutants and gradually accumulate within ecosystems. Although existing studies have documented MP presence in multiple terrestrial and aquatic environments, the cryosphere remains comparatively understudied. To comprehensively understand the extent of MP pollution, future research efforts should prioritize unexplored cryosphere regions (e.g., the Himalayan cryosphere). Additionally, the impacts of MP on radiation balance, snow/ice melt dynamics, and glacier shrinkage, including effects on the surface-energy balance, need to be further investigated.

The sources, pathways, and mechanisms of transport of these particles, considering geographical and meteorological conditions, need to be studied. Ice cores need to be drilled to study the historical temporal trends of MPs in these environments. To address current challenges and uncertainties, future perspectives in MP research in glacial environments should involve in-depth assessments on the environmental impacts of these MPs, including effects on organisms, trophic transfer along food webs, interactions with microorganisms, impacts on microorganisms, and atmosphere-radiative influences. In addition, standardized monitoring methods need to be established to ensure reliability and data comparability across global cryospheric studies. There is also a need for international collaboration to develop consistent guidelines and assessment frameworks for MP investigations in glacial environments. Furthermore, global policy-oriented efforts for reducing plastic waste loads should be considered, particularly for regions reporting disproportionately high plastic waste generation. These efforts are essential for preserving the cryospheric ecosystem integrity and for understanding the broader influences of MP on climate interactions and high-mountain biota.

In addition to increasing observational studies, there is now a clear need to shift toward understanding the mechanisms and future behavior of MPs in the cryosphere. This shift entails addressing interconnected challenges, including method standardization, quantification of transport pathways, environmental interactions, ecological risk, and long-term prediction. Addressing these issues requires interdisciplinary collaboration across glaciology, environmental science, toxicology, and atmospheric sciences. Key future priorities include developing reliable nanoplastic-detection techniques, refining high-resolution atmospheric and glacial transport models, experimentally simulating freeze-thaw and photodegradation processes, and creating biotoxicity databases tailored to polar and alpine ecosystems. These integrated efforts are essential for providing a robust scientific foundation to support the protection and management of cryospheric environments in a changing climate.

## Conclusion

This review summarizes current knowledge of microplastic contamination in the cryosphere, demonstrating widespread occurrence in glaciers, snow, and sea ice. Fine microplastic particles, notably PET, PE, PP, and PA, reach cryospheric environments via both long-range atmospheric transport and local-scale sources. Cryospheric compartments act as temporary sinks and may serve as secondary sources of downstream contamination through meltwater export. Beyond ecological exposure, dark and fine microplastics can reduce the surface albedo and contribute to enhanced melt, linking microplastic deposition on snow and ice to regional climate feedbacks and warming-amplification pathways. Significant knowledge gaps remain in the harmonized sampling and analytical protocols, high-resolution temporal records, and the understanding of particle fate and ecological impacts. Addressing these gaps through standardized methods, long-term monitoring, targeted source-apportionment studies, and integrated process-based modeling is essential to quantify environmental and climate-related consequences and to guide the development of mitigation strategies.

## Acknowledgments

Faisal Zahoor Jan expresses gratitude for the financial support received from the 10.13039/501100001409Department of Science and Technology, Government of India (DST, GoI), for the INSPIRE fellowship [Grant ID: IF220404] for his Ph.D. studies. Irfan Rashid acknowledges the Jammu and Kashmir Science, Technology, and Innovation Council for Research Grant [Grant ID: JKST&IC/SRE/160-164]. Chandan Sarangi and Irfan Rashid acknowledge the 10.13039/501100001851Ministry of Earth Sciences, Government of India, for the Research Grant under the EU-Horizon project [Grant ID: MoES/EU/HorizonEurope/1/24-PC-I]. Gulzar A. Bhat gratefully acknowledges 10.13039/501100001409DST-SERB for the Core Research Grant [Grant ID: CRG/2022/003558] and J&K Science Technology & Innovation Council [Grant ID: JKST&IC/SRE/90-94]. Gulzar A. Bhat also acknowledges the DST PURSE Grant-supported central facility. All the authors gratefully acknowledge the Associate Scientific Editor, Dr. Avinash Alagumalai, and the two anonymous reviewers for their insightful feedback and constructive comments, which enhanced the quality, structure, and clarity of the article.

## Author contributions

I.R. and G.A.B. conceived the article. I.Q., F.Z.H., I.R., and G.A.B. wrote the first draft of the article. I.R., G.A.B., A.A., and C.S. revised the article.

## Declaration of interests

The authors declare no competing interests.
