# Supplementary material for: A review of microplastic contamination in the cryosphere
Source: iScience. 2025 Dec 13;29(1):114414. doi: 10.1016/j.isci.2025.114414 (PMC12808910; doi:10.1016/j.isci.2025.114414)
Supplement: Document S1. Supplemental methods [file mmc1.pdf]

iScience, Volume *29*

Supplemental information

A review of microplastic contamination in the cryosphere

Irteza Qayoom, Faisal Zahoor Jan, Irfan Rashid, Gulzar A. Bhat, Anoop Ambili, and Chandan Sarangi

SUPPLEMENTARY MATERIAL FOR

Microplastic pollution in the cryosphere: a review of sources, identification, distribution, and environmental impacts

Irteza Qayoom [a], Faisal Zahoor Jan [b], Irfan Rashid [b]*, Gulzar A. Bhat [a, c]**, Anoop Ambili [d], Chandan Sarangi [e]

Author affiliations:

[a] Centre for Interdisciplinary Research and Innovations, University of Kashmir, Srinagar, 190006, Jammu and Kashmir, India

[b] Department of Geoinformatics, University of Kashmir, Srinagar, 190006, Jammu and Kashmir, India

[c] Department of Chemistry, University of Kashmir, Srinagar, 190006, Jammu and Kashmir, India

[d] Department of Earth and Environmental Sciences, Indian Institute of Science Education & Research Mohali, Mohali, 140306, Punjab, India

[e] Department of Civil Engineering, Indian Institute of Technology Madras, Chennai, 600036, Tamil Nadu, India

IQ and FZJ have equal contributions

Corresponding authors' email:

Irfan Rashid (*): irfangis@kashmiruniversity.ac.in

Gulzar A. Bhat (**): gulzarbhat@uok.edu.in

METHODS FOR IDENTIFYING MPs IN THE CRYOSPHERE

The identification and quantification of MP in cryospheric environments present unique challenges due to their small size [1,2], low MP concentration [3,4], complex matrices (ice-sediment mixtures) [5,6], contamination risks in remote regions, and the often inaccessible nature of these regions [7,8]. To minimize potential problems and ensure accurate and reliable results, careful sampling techniques, clean equipment, and minimizing self-contamination from clothing are crucial, as they can serve as a significant source accounting for up to 15% of MP fibres [9]. Various methods have been employed to detect and characterize MPs, each with its advantages and limitations. Overall, the procedure can be broadly categorized into sampling, density separation, and analysis [10,11].

Sampling

Sampling involves extracting layers at specific depths from random locations. Surface snow provides valuable data about recent depositions, whereas glacial ice cores offer insights into historical contamination levels. Snow sampling is carried out by collecting surface snow to a specific depth using a pre-cleaned spoon, shovel, or scoop [12] then transferred to cleaned containers (glass, metal, or plastic bags washed with Milli-Q water, acetone, or ethanol) (18 MΩ) [13,14,15].

Pre-treatment

This involves a sequence of procedures such as density separation, filtration, and drying to concentrate, purify, and prepare samples for accurate identification and quantification (**Table 1**). The density separation technique separates MPs from other particles based on their densities, purifies the sample, and removes any contaminants. Different salts such as sodium chloride (NaCl), calcium chloride ($CaCl_2$), zinc bromide ($ZnBr_2$), sodium bromide (NaBr), and sodium iodide (NaI) have been used for centrifugation by the scientific community. The sample is then digested by using strong acids or alkalis to dissolve impurities in the sample, leaving behind pure MPs. Different chemicals such as nitric acid (HNO_3), potassium hydroxide (KOH), sodium hydroxide (NaOH), hydrochloric acid (HCl), hydrogen peroxide (H_2O_2), and a combination of hydrogen peroxide and sulfuric acid (H_2O_2 + H_2SO_4), Fenton's Reagent (H_2O_2 + Fe^{2+}), and enzymes have been used for the digestion of impurities [16-18]. For cryospheric studies, labile organic material adsorbed on the MPs is removed through wet peroxide oxidation (WPO), digestion [19,2], enzymes like Creon [20], and different concentrations of H_2O_2 solution (15% for sediment samples

and 35% for ice core samples) [8,21,2,14,22] since organic-rich samples interfere with the resulting spectra [23,24] (**Table 1**).

The filtration process involves using micro-sized filters to separate contaminants and other particles from the snow sample, leaving MPs for analysis. Different filters with varying pore sizes have been used for the filtration process, including glass fibre filters [25,3,26,27,28,29], nitrocellulose filters [5,6,30,14,15], polyethylene terephthalate (PET) filters [31], aluminium oxide filters [8,32,2], paper fibre filter disks [33,20], and polycarbonate gold-coated membrane filters [12] (**Table 1**). Glass microfiber filters are commonly utilized for glacier samples owing to their ability to effectively filter out small particles, while cellulose nitrate filters are also preferred at times for their availability and affordability [10]. After filtration, MPs are retained onto the filter, and the filtrate is rinsed with 10–20 mL of 70 % ethanol, and a further 10-30 mL of 96 % ethanol to eliminate any bacteria or viruses. After the sample filtration, the sides of the glassware are rinsed with ultra-pure water to remove any MPs that may have adhered to the walls of the filtering equipment. The filtered sample is then dried under a vacuum to remove any remaining moisture. Then the MPs on the filter are analyzed using different techniques, such as microscopy and spectroscopy, to identify and quantify the particle concentration.

Characterization

Various techniques have been utilized for identifying MP in snow and ice [21,34-36] and are broadly categorized into physical and chemical approaches (for details, see **Table 1**). Physical methods involve the direct analysis of the physical attributes of MPs, including size (length, width, and texture), number, shape, and color [32,30,29,37]. Stereomicroscopes are commonly used for this purpose [38,27,30]. Stereomicroscopes identify MPs with a particle size within the range of 1-5 mm [39]. Scanning Electron Microscope (SEM) is also used for MP identification and, in combination with Energy-dispersive X-ray spectroscopy (SEM-EDS), further provides information on the elemental composition and micromorphology. For enhanced visual assessment, MPs are stained with fluorescent dyes, particularly Nile Red (NR), to enhance identification using optical microscopy. This allows efficient identification of MP ranging from 3-300 μm [40,41]. However, staining efficiency can vary depending on the polymer shape, type, and solvent used, making robust polymer identification challenging.

Spectroscopic techniques (chemical approach) such as Fourier-Transform InfraRed spectroscopy (FTIR) and Raman spectroscopy [42-44] are most commonly employed in the studies

for identification (shape and polymer type) and quantification (size distribution) of MP. These methods are non-destructive and require a small amount (a few microlitres or a single particle) of samples. FTIR provides information about the chemical bonds and functional groups of a sample by analyzing the infrared spectrum of the sample [34,43]. FTIR with rich spectral libraries can identify MP with a minimum size of ~10 μm [45]. However, for MP <50 μm FTIR may underestimate the quantity in a sample [43,46-48]. Raman spectroscopy has also been used to identify polymer types in cryosphere studies [30,12,33,49,42]. This technique determines the particle count, size distribution, and morphological characteristics with better resolution (down to 1 μm) [43,50,51], however, it is costly and requires more time, and is prone to interference from fluorescence. This will obscure the characteristic Raman peaks of the MP, making it difficult to distinguish them from background fluorescence signal, potentially leading to false negatives or misinterpretations of data, especially when analyzing small or coloured MPs with high levels of fluorescent additives [52]. Sometimes, weathered plastic can interfere with Raman signals, yielding different values compared to unweathered plastic [53]. Micro-Raman (μ-Raman) is also applicable for visual observation and offers insights into particle size distribution, quantity, and morphological characteristics. Another method, thermal desorption-proton transfer reaction mass spectrometry (TD-PTR-MS), has also been employed to detect various types of nano-plastics in 0.2 μm-filtered water samples sourced from snow in the Austrian Alps [31]. This technique allows the identification of the chemical species that make up polymers at the level of the chemical formula. TD-PTR-MS has a high sensitivity (<1 ng) and can provide quantitative information on low-concentration organics (sample volume 1 mL), making it the most sensitive and precise method for identifying environmental MPs [31].

Pyrolysis-gas chromatography-mass spectrometry (Py-GC-MS) is also an effective technique for the characterization of MPs, such as polystyrene, polyethylene, and polypropylene [24,43]. This technique involves thermal decomposition of samples across one or multiple temperature ranges and enables polymer identification through analysis of distinctive degradation products [54]. The use of an additional thermal desorption step allows for the identification of GC peaks corresponding to additives or sorbed hydrophobic organic pollutants and is not limited by particle size, making it a promising technique for the analysis of microplastics and nanoplastics (MNP) [55,56]. Recently, a method has been developed for the picogram-level quantification of MNP in water and snow matrices using in-laboratory synthesized nanostructures (titanium oxide, zinc oxide, and cobalt) coupled with mass spectrometry techniques, without the need for sample pre-treatment. The method detects micro- and nano-plastics at levels as low as approximately 5 picograms in

ambient snow. The development of an ultra-trace quantification method for micro- and nano-plastics is based on nanostructured laser desorption/ionization time-of-flight mass spectrometry (NALDI-TOF-MS) [57]. Quantum cascade laser infrared (QCL-IR) spectroscopy has also gained attention for its rapid analytical performance [41]. Additionally, atomic force microscopy-based infrared (AFM-IR) spectroscopy has enabled nanoscale analysis, advancing the boundaries of MNP characterization [58]. MPs are also analyzed using Laser Direct Infrared Spectroscopy (LDIR), an advanced technique that quickly identifies polymer types by targeting the fingerprint region of the infrared spectrum, where molecules exhibit distinct vibrational patterns [59].

Further advancements in instrumentation are crucial for improving the accuracy and efficiency of MP analysis. The development of novel infrared (IR) spectroscopic instruments with enhanced spatial resolution and sensitivity is particularly important. Additionally, increasing emphasis is being placed on miniaturization and portability, enabling on-site detection and real-time monitoring of MPs across diverse environmental matrices. The integration of machine learning algorithms into data analysis workflows presents a promising avenue for improving the efficiency, accuracy, and automation of MP identification and classification. Furthermore, standardization and quality assurance efforts will be critical in ensuring the reliability, reproducibility, and comparability of results across different studies.

Quality Assurance/ Quality Control (QA/QC)

Establishing QA/QC approaches during the sample collection, transport, extraction, and MP analysis in laboratory settings is essential to enhancing data reliability. Till now, there are no standardized approaches, although some researchers have taken individual measures for QA/QC, such as replication level, distance between sampling sites, and measures for controlling contamination. In addition, all laboratory procedures need to be carried out in a dedicated MP clean laboratory, purpose-built to meet the necessary standards. The laboratory must possess a positive-pressure air system, controlled access, and appropriate procedural blanks. Negative (includes field controls collected simultaneously with sampling and laboratory controls) and positive (by adding amounts of MP) controls should be collected, treated, and analyzed alongside samples. Working solutions, which include solvents and reagents, must be pre-filtered during sample processing. Cotton laboratory coats should be used to reduce the contamination risk from synthetic fabrics [5]. Additionally, all laboratory apparatus should be made of either glass or stainless steel and thoroughly rinsed with filtered 1.6 mm Milli-Q water before use [27] taking into

consideration that detailed reporting of particle characterization, including shape, size, and chemical composition.

References

1. Wen, H., Xu, H., Ma, Y., Zhang, C., Zhang, D., and Wang, X. (2024). Diverse and high pollution of microplastics in seasonal snow across Northeastern China. Science of the Total Environment *907*, 167923.
2. Peeken, I., Primpke, S., Beyer, B., Gütermann, J., Katlein, C., Krumpen, T., Bergmann, M., Hehemann, L., and Gerdts, G. (2018). Arctic sea ice is an important temporal sink and means of transport for microplastic. Nature Communications *9*, 1505.
3. Von Friesen, L. W., Granberg, M. E., Pavlova, O., Magnusson, K., Hassellöv, M., & Gabrielsen, G. W. (2020). Summer sea ice melt and wastewater are important local sources of microlitter to Svalbard waters. Environment International, *139*, 105511.
4. Materić, D., Kjær, H.A., Vallelonga, P., Tison, J.-L., Röckmann, T., and Holzinger, R. (2022). Nanoplastics measurements in Northern and Southern polar ice. Environmental Research *208*, 112741.
5. Obbard, R.W., Sadri, S., Wong, Y.Q., Khitun, A.A., Baker, I., and Thompson, R.C. (2014). Global warming releases microplastic legacy frozen in Arctic Sea ice. Earth's Future *2*, 315-320.
6. Crosta, A., De Felice, B., Antonioli, D., Chiarcos, R., Perin, E., Ortenzi, M.A., Gazzotti, S., Azzoni, R.S., Fugazza, D., and Gianotti, V. (2022). Microplastic contamination of supraglacial debris differs among glaciers with different anthropic pressures. Science of the Total Environment *851*, 158301.
7. YÜCEL, N., TUTSAK, E., and KILIÇ, E. (2023). First evidence of microplastic deposition in snow from Turkey. Journal of Anatolian Environmental and Animal Sciences *8*, 95-102.
8. Kelly, A., Lannuzel, D., Rodemann, T., Meiners, K., and Auman, H. (2020). Microplastic contamination in east Antarctic sea ice. Marine Pollution Bulletin *154*, 111130.
9. Scopetani, C., Esterhuizen-Londt, M., Chelazzi, D., Cincinelli, A., Setälä, H., and Pflugmacher, S. (2020). Self-contamination from clothing in microplastics research. Ecotoxicology and Environmental Safety *189*, 110036.
10. Cabrera, M., Valencia, B.G., Lucas-Solis, O., Calero, J.L., Maisincho, L., Conicelli, B., Moulatlet, G.M., and Capparelli, M.V. (2020). A new method for microplastic sampling and isolation in mountain glaciers: A case study of one antisana glacier, Ecuadorian Andes. Case Studies in Chemical and Environmental Engineering *2*, 100051.
11. Enyoh, C.E., Verla, A.W., Verla, E.N., Ibe, F.C., and Amaobi, C.E. (2019). Airborne microplastics: a review study on method for analysis, occurrence, movement and risks. Environmental Monitoring and Assessment *191*, 668.
12. Wang, Z., Zhang, Y., Kang, S., Yang, L., Luo, X., Chen, P., Guo, J., Hu, Z., Yang, C., and Yang, Z. (2022). Long-range transport of atmospheric microplastics deposited onto glacier in southeast Tibetan Plateau. Environmental Pollution *306*, 119415.
13. González-Pleiter, M., Edo, C., Velázquez, D., Casero-Chamorro, M.C., Leganés, F., Quesada, A., Fernández-Piñas, F., and Rosal, R. (2020). First detection of microplastics in the freshwater of an Antarctic Specially Protected Area. Marine Pollution Bulletin *161*, 111811.
14. Parolini, M., Antonioli, D., Borgogno, F., Gibellino, M.C., Fresta, J., Albonico, C., De Felice, B., Canuto, S., Concedi, D., and Romani, A. (2021). Microplastic contamination in snow from Western Italian Alps. International Journal of Environmental Research and Public Health *18*, 768.
15. Cabrera, M., Moulatlet, G.M., Valencia, B.G., Maisincho, L., Rodríguez-Barroso, R., Albendín, G., Sakali, A., Lucas-Solis, O., Conicelli, B., and Capparelli, M.V. (2022). Microplastics in a tropical

Andean Glacier: A transportation process across the Amazon basin? Science of the Total Environment *805*, 150334.

16. Hanvey, J.S., Lewis, P.J., Lavers, J.L., Crosbie, N.D., Pozo, K., and Clarke, B.O. (2017). A review of analytical techniques for quantifying microplastics in sediments. Analytical Methods *9*, 1369-1383.

17. Löder, M.G., Imhof, H.K., Ladehoff, M., Löschel, L.A., Lorenz, C., Mintenig, S., Piehl, S., Primpke, S., Schrank, I., and Laforsch, C. (2017). Enzymatic purification of microplastics in environmental samples. Environmental Science & Technology *51*, 14283-14292.

18. Renner, G., Schmidt, T.C., and Schram, J. (2018). Analytical methodologies for monitoring micro (nano) plastics: which are fit for purpose? Current Opinion in Environmental Science & Health *1*, 55-61.

19. Aves, A.R., Revell, L.E., Gaw, S., Ruffell, H., Schuddeboom, A., Wotherspoon, N.E., LaRue, M., and McDonald, A.J. (2022). First evidence of microplastics in Antarctic snow. The Cryosphere *16*, 2127-2145.

20. Pastorino, P., Pizzul, E., Bertoli, M., Anselmi, S., Kušće, M., Menconi, V., Prearo, M., and Renzi, M. (2021). First insights into plastic and microplastic occurrence in biotic and abiotic compartments, and snow from a high-mountain lake (Carnic Alps). Chemosphere *265*, 129121.

21. Zhang, Y., Gao, T., Kang, S., Shi, H., Mai, L., Allen, D., and Allen, S. (2022). Current status and future perspectives of microplastic pollution in typical cryospheric regions. Earth-Science Reviews *226*, 103924.

22. Ambrosini, R., Azzoni, R.S., Pittino, F., Diolaiuti, G., Franzetti, A., and Parolini, M. (2019). First evidence of microplastic contamination in the supraglacial debris of an alpine glacier. Environmental Pollution *253*, 297-301.

23. Löder, M.G., and Gerdts, G. (2015). Methodology used for the detection and identification of microplastics—a critical appraisal. Marine Anthropogenic Litter, 201-227.

24. Zhang, X., Zhang, H., Yu, K., Li, N., Liu, Y., Liu, X., Zhang, H., Yang, B., Wu, W., and Gao, J. (2020). Rapid monitoring approach for microplastics using portable pyrolysis-mass spectrometry. Analytical Chemistry *92*, 4656-4662.

25. Geilfus, N.-X., Munson, K., Sousa, J., Germanov, Y., Bhugaloo, S., Babb, D., and Wang, F. (2019). Distribution and impacts of microplastic incorporation within sea ice. Marine Pollution Bulletin *145*, 463-473.

26. Villanova-Solano, C., Hernández-Sánchez, C., Díaz-Peña, F.J., González-Sálamo, J., González-Pleiter, M., and Hernández-Borges, J. (2023). Microplastics in snow of a high mountain national park: El Teide, Tenerife (Canary Islands, Spain). Science of the Total Environment *873*, 162276.

27. Napper, I.E., Davies, B.F., Clifford, H., Elvin, S., Koldewey, H.J., Mayewski, P.A., Miner, K.R., Potocki, M., Elmore, A.C., and Gajurel, A.P. (2020). Reaching new heights in plastic pollution—preliminary findings of microplastics on Mount Everest. One Earth *3*, 621-630.

28. Malygina, N., Biryukov, R.Y., Kuryatnikova, N., Mitrofanova, E.Y., Pershin, D., Zolotov, D., and Chernykh, D. (2020). Microplastics in the snow cover of the south of Western Siberia. In 1. (IOP Publishing), 611, pp. 012034.

29. Kanhai, L.D.K., Gardfeldt, K., Krumpen, T., Thompson, R.C., and O'Connor, I. (2020). Microplastics in sea ice and seawater beneath ice floes from the Arctic Ocean. Scientific Reports *10*, 5004.

30. Stefánsson, H., Peternell, M., Konrad-Schmolke, M., Hannesdóttir, H., Ásbjörnsson, E.J., and Sturkell, E. (2021). Microplastics in glaciers: first results from the Vatnajökull ice cap. Sustainability *13*, 4183.

31. Materić, D.a., Kasper-Giebl, A., Kau, D., Anten, M., Greilinger, M., Ludewig, E., van Sebille, E., Röckmann, T., and Holzinger, R. (2020). Micro-and nanoplastics in alpine snow: a new method for

chemical identification and (semi) quantification in the nanogram range. Environmental science & technology *54*, 2353-2359.
32. Bergmann, M., Mützel, S., Primpke, S., Tekman, M.B., Trachsel, J., and Gerdts, G. (2019). White and wonderful? Microplastics prevail in snow from the Alps to the Arctic. Science advances *5*, eaax1157.
33. Zhang, Y., Gao, T., Kang, S., Allen, S., Luo, X., and Allen, D. (2021). Microplastics in glaciers of the Tibetan Plateau: evidence for the long-range transport of microplastics. Science of the Total Environment *758*, 143634.
34. Hendrickson, E., Minor, E.C., and Schreiner, K. (2018). Microplastic abundance and composition in western Lake Superior as determined via microscopy, Pyr-GC/MS, and FTIR. Environmental Science & Technology *52*, 1787-1796.
35. Mai, L., Bao, L.-J., Shi, L., Wong, C.S., and Zeng, E.Y. (2018). A review of methods for measuring microplastics in aquatic environments. Environmental Science and Pollution Research *25*, 11319-11332.
36. Wagner, M., and Lambert, S. (2018). Freshwater microplastics: emerging environmental contaminants? Springer Nature,303.
37. Hidalgo-Ruz, V., Gutow, L., Thompson, R.C., and Thiel, M. (2012). Microplastics in the marine environment: a review of the methods used for identification and quantification. Environmental Science & Technology *46*, 3060-3075.
38. Primpke, S., Booth, A.M., Gerdts, G., Gomiero, A., Kögel, T., Lusher, A., Strand, J., Scholz-Böttcher, B.M., Galgani, F., and Provencher, J. (2022). Monitoring of microplastic pollution in the Arctic: recent developments in polymer identification, quality assurance and control, and data reporting. Arctic Science *9*, 176-197.
39. Primpke, S., Christiansen, S.H., Cowger, W., De Frond, H., Deshpande, A., Fischer, M., Holland, E.B., Meyns, M., O'Donnell, B.A., and Ossmann, B.E. (2020). Critical assessment of analytical methods for the harmonized and cost-efficient analysis of microplastics. Applied Spectroscopy *74*, 1012-1047.
40. Araujo, C.F., Nolasco, M.M., Ribeiro, A.M., and Ribeiro-Claro, P.J. (2018). Identification of microplastics using Raman spectroscopy: Latest developments and future prospects. Water Research *142*, 426-440.
41. Käppler, A., Fischer, D., Oberbeckmann, S., Schernewski, G., Labrenz, M., Eichhorn, K.-J., and Voit, B. (2016). Analysis of environmental microplastics by vibrational microspectroscopy: FTIR, Raman or both? Analytical and Bioanalytical Chemistry *408*, 8377-8391.
42. Primpke, S., Wirth, M., Lorenz, C., and Gerdts, G. (2018). Reference database design for the automated analysis of microplastic samples based on Fourier transform infrared (FTIR) spectroscopy. Analytical and Bioanalytical Chemistry *410*, 5131-5141.
43. Cowger, W., Roscher, L., Jebens, H., Chamas, A., Maurer, B.D., Gehrke, L., Gerdts, G., and Primpke, S. (2024). Generation of macro-and microplastic databases by high-throughput FTIR analysis with microplate readers. Analytical and Bioanalytical Chemistry *416*, 1311-1320.
44. Dris, R., Gasperi, J., Mirande, C., Mandin, C., Guerrouache, M., Langlois, V., and Tassin, B. (2017). A first overview of textile fibers, including microplastics, in indoor and outdoor environments. Environmental Pollution *221*, 453-458.
45. Li, X., Ding, Y., Xu, J., He, X., Han, T., Kang, S., Wu, Q., Mika, S., Yu, Z., and Li, Q. (2018). Importance of mountain glaciers as a source of dissolved organic carbon. Journal of Geophysical Research: Earth Surface *123*, 2123-2134.
46. Liu, K., Wang, X., Fang, T., Xu, P., Zhu, L., and Li, D. (2019). Source and potential risk assessment of suspended atmospheric microplastics in Shanghai. Science of the Total Environment *675*, 462-471.

47. Allen, S., Allen, D., Phoenix, V.R., Le Roux, G., Durántez Jiménez, P., Simonneau, A., Binet, S., and Galop, D. (2019). Atmospheric transport and deposition of microplastics in a remote mountain catchment. Nature Geoscience *12*, 339-344.
48. Anger, P.M., von der Esch, E., Baumann, T., Elsner, M., Niessner, R., and Ivleva, N.P. (2018). Raman microspectroscopy as a tool for microplastic particle analysis. TrAC Trends in Analytical Chemistry *109*, 214-226.
49. Huppertsberg, S., and Knepper, T.P. (2018). Instrumental analysis of microplastics—benefits and challenges. Analytical and Bioanalytical Chemistry *410*, 6343-6352.
50. Azari, A., Ronsmans, S., Vanoirbeek, J.A., Hoet, P.H., and Ghosh, M. (2024). Challenges in Raman spectroscopy of (micro) Plastics: The interfering role of colourants. Environmental Pollution *363*, 125250.
51. Phan, S., Padilla-Gamiño, J.L., and Luscombe, C.K. (2022). The effect of weathering environments on microplastic chemical identification with Raman and IR spectroscopy: Part I. polyethylene and polypropylene. Polymer Testing *116*, 107752.
52. Picó, Y., and Barceló, D. (2020). Pyrolysis gas chromatography-mass spectrometry in environmental analysis: Focus on organic matter and microplastics. TrAC Trends in Analytical Chemistry *130*, 115964.
53. Mintenig, S., Bäuerlein, P.S., Koelmans, A.A., Dekker, S.C., and Van Wezel, A. (2018). Closing the gap between small and smaller: towards a framework to analyse nano-and microplastics in aqueous environmental samples. Environmental Science: Nano *5*, 1640-1649.
54. Ter Halle, A., Jeanneau, L., Martignac, M., Jardé, E., Pedrono, B., Brach, L., and Gigault, J. (2017). Nanoplastic in the North Atlantic subtropical gyre. Environmental Science & Technology *51*, 13689-13697.
55. Wang, Z., Saadé, N.K., and Ariya, P.A. (2021). Advances in ultra-trace analytical capability for micro/nanoplastics and water-soluble polymers in the environment: fresh falling urban snow. Environmental Pollution *276*, 116698.
56. Xie, J., Gowen, A., Xu, W., and Xu, J. (2024). Analysing micro-and nanoplastics with cutting-edge infrared spectroscopy techniques: a critical review. Analytical Methods, 16, 2177-2197.
57. Ourgaud, M., Phuong, N.N., Papillon, L., Panagiotopoulos, C., Galgani, F., Schmidt, N., Fauvelle, V., Brach-Papa, C., and Sempéré, R. (2022). Identification and quantification of microplastics in the marine environment using the laser direct infrared (LDIR) technique. Environmental Science & Technology *56*, 9999-10009.
58. Chun, S., Gopal, J., and Muthu, M. (2024). Portable analytics as a contemporary environmental microplastic research tool: The Pitches and Hitches. Trends in Environmental Analytical Chemistry, e00234.
59. Wright, S.L., Gouin, T., Koelmans, A.A., and Scheuermann, L. (2021). Development of screening criteria for microplastic particles in air and atmospheric deposition: critical review and applicability towards assessing human exposure. Microplastics and Nanoplastics *1*, 6.
